# Exergoeconomic, Exergoenvironmental, and Renewability Assessment of Corn Stover and Sugarcane Bagasse PLA Biorefineries With Co‐Product Valorization

**DOI:** 10.1002/gch2.70164

**Published:** 2026-10-24

**Authors:** Reza Hessampour, Ali Jahanian, Jerome Ramirez, Ian O'Hara

**Affiliations:** ^1^ School of Mechanical Medical and Process Engineering Faculty of Engineering Queensland University of Technology Brisbane Australia; ^2^ Centre for Agriculture and Bioeconomy Faculty of Science Queensland University of Technology Brisbane Australia; ^3^ ARC Industrial Transformation Training Centre for Bioplastics and Biocomposites Queensland University of Technology Brisbane Australia; ^4^ ARC Centre of Excellence in Synthetic Biology Queensland University of Technology Brisbane Australia

**Keywords:** co‐product integration, exergoeconomic analysis, exergoenvironmental analysis, exergy analysis, polylactic acid (PLA) biorefinery

## Abstract

Lignocellulosic polylactic acid biorefineries face challenges due to high energy demand, complex downstream processing, and underutilized co‐products. Although lignocellulosic biomass is a promising feedstock, the sustainability, and economic impacts of integrating multiple value‐added products remain unclear. This study develops an integrated thermodynamic, exergoeconomic, exergoenvironmental, and renewability assessment of bagasse‐ and corn stover‐based biorefineries producing polylactic acid, electricity, and co‐products including succinic acid, xylitol, and adhesives. Sugarcane bagasse exhibits higher fuel exergy input (103.94–138.47 GJ per functional unit (FU)) than corn stover (97.41–128.49 GJ/FU), with marginally higher product exergy output. Overall exergy efficiencies remain comparable (44%–64% for corn stover and 47%–65% for bagasse). Electricity‐integrated configurations show poorer thermodynamic performance, with exergy destruction exceeding 55 GJ/FU, whereas adhesive‐integrated configurations reduce destruction to 45.45–50.75 GJ/FU (corn stover) and 47.39–47.72 GJ/FU (bagasse), achieving efficiencies up to 65%. Exergoeconomic results indicate that downstream purification dominates cost formation. Succinic acid purification costs increase from 344.19 to 958.85 USD/GJ (corn stover to bagasse), while xylitol purification reaches 566.57 and 288.04 USD/GJ, respectively. Exergoenvironmental analysis identifies purification and pretreatment as hotspots. Renewability analysis shows that electricity‐based configurations remain non‐renewable (renewability indicator (RI)<0), whereas adhesive‐integrated scenarios achieve partial renewability (RI = 0.35–0.47). Overall, co‐product strategy and downstream processing influence system performance more strongly than feedstock selection.

## Graphical abstract

Which matters more for sustainable polylactic acid production, feedstock, or co‐product choice? Comparing corn stover and sugarcane bagasse biorefineries, this study shows that co‐product strategy dominates: electricity‐integrated pathways stay thermodynamically inefficient and non‐renewable, while adhesive‐integrated configurations reach 65% exergy efficiency and partial renewability, with downstream purification driving costs and environmental impact.

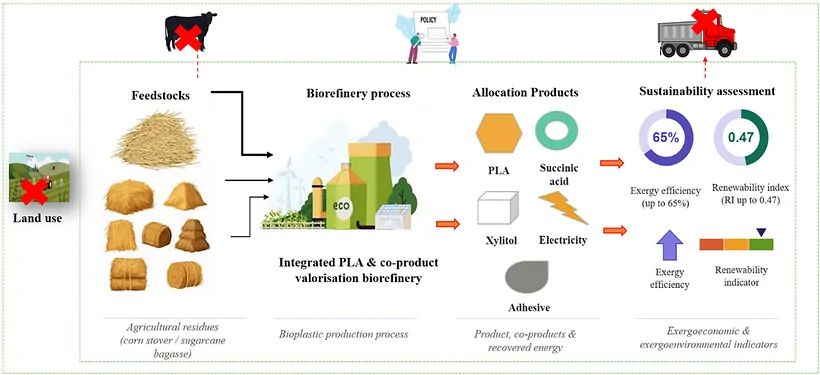

## 1 Introduction

Due to the rapid increase in plastic production and usage worldwide, plastics have become persistent environmental contaminants [1]. The plastics industry remains economically important due to its capacity to generate revenue and supply a wide range of products across multiple sectors [2]. However, global production has exceeded 300 Million metric tonnes annually and continues to grow, with projections indicating further increases in oil demand if this trend persists [3]. The environmental consequences associated with plastic waste are significant, as it can persist for thousands of years and release harmful substances into soil and water systems during degradation [4, 5]. These challenges have intensified the need for alternative materials that can mitigate the environmental impacts associated with conventional plastics.

In response to these environmental challenges, the development of biodegradable plastics that can be directly disposed of in soil and degrade within a specified timeframe has gained increasing attention [6]. Polylactic acid (PLA) is recognized as a promising alternative to petrochemical‐based plastics [7]. PLA is a thermoplastic that naturally decomposes and can be shaped into a variety of bio‐based products [8]. PLA production requires L‐lactic acid (LLA) as its monomer, which can be produced through the fermentation of glucose and xylose [9]. These fermentable sugars can be obtained from biomass‐derived feedstocks through pretreatment and hydrolysis [10]. Although edible crops can be used as sugar sources, their utilization for bioplastic production may contribute to food price inflation [11]. In contrast, lignocellulosic biomass is considered an attractive alternative feedstock for PLA production because it is renewable, abundant, and relatively low‐cost, offering a more sustainable option than fossil resources and starch‐based biomass traditionally used for lactic acid production [12]. Lignocellulosic resources include agricultural residues such as corn stover, sugarcane bagasse, sorghum stalks, and cow manure, supporting a non‐food biorefinery concept that reduces competition with food resources and improves the environmental sustainability of plastic production [13]. Within this context, biorefineries provide an integrated platform for converting biomass‐derived feedstocks into PLA and other value‐added products.

The ability of biorefineries to convert biomass into a diverse range of commercially viable products is garnering increasing attention [14] due to its potential for sustainability, economic benefits, and reduced environmental impact [15, 16]. Biomass offers a competitive edge in developing refining technologies, particularly due to the ability to utilize waste material streams in industrial processes. This approach is central to establishing a sustainable circular economy and transitioning away from a fossil fuel‐based system [17, 18, 19]. Sustainability is a crucial element in the creation and operation of biorefineries, fundamentally shaping their development, and strategic significance [20]. The biorefinery concept is intrinsically connected to the principles of a circular bioeconomy, which seeks to minimize environmental impact through the efficient and sustainable utilization of biological resources [21]. Accordingly, robust analytical methods are required to evaluate the efficiency and sustainability of such integrated biorefinery systems.

Several methodologies have been employed to assess system efficiency and environmental sustainability, including life cycle assessment [10, 22, 23], thermo‐economic analysis [24, 25], techno‐economic analysis [26, 27, 28, 29], energy analysis [10], and energy‐economic analysis [30]. Among these, exergy analysis is a thermodynamic method that combines the first and second laws of thermodynamics to assess both energy quality and process efficiency [31]. It quantifies the maximum useful work obtainable as a system moves from its current state to equilibrium with the environment [32]. By evaluating the quantity and quality of energy and material flows, exergy analysis enables precise identification of the sources, locations, and types of thermodynamic inefficiencies (irreversibilities) in energy conversion systems. In practical processes, exergy is unavoidably degraded through irreversibilities, and analysing these losses helps identify system waste patterns [33]. After identifying the main sources of thermodynamic irreversibility, targeted improvements can be implemented to enhance system efficiency. Accordingly, exergy analysis provides deeper insight than energy analysis by accounting for both energy quantity and quality and by quantifying irreversibilities.

Previous techno‐economic (TEA) and life‐cycle assessment (LCA) studies have evaluated polylactic acid (PLA) production from a range of first‐ and second‐generation feedstocks, including sugarcane and waste‐derived biorefinery systems [34, 35, 36], corn and sugarcane molasses [37], sugar beet pulp [36, 38], corn stover [39, 40], and bamboo residue [41]. More recently, artificial intelligence‐assisted frameworks have been applied to quantify uncertainty and variability in the techno‐economic and life‐cycle performance of corn stover‐derived PLA [42, 43], reflecting a growing emphasis on robust, data‐driven assessment of second‐generation PLA biorefineries. Complementary exergy‐based sustainability assessments have also been applied to agricultural and agro‐industrial systems [44, 45].

Despite increasing interest in lignocellulosic PLA biorefineries, cost competitiveness remains a major barrier to industrial deployment, particularly in single‐product configurations. Although co‐product integration has been proposed as a strategy to improve economic viability, the relative influence of feedstock selection and co‐product portfolio design on thermodynamic, economic, and environmental performance remains poorly understood. To date, no study has systematically compared corn stover and sugarcane bagasse‐based PLA biorefineries within a unified exergy‐based framework while simultaneously evaluating alternative co‐product pathways, including succinic acid, xylitol, lignin‐based adhesive, and electricity generation. These four co‐product options were selected to identify which valorization strategy could most improve the economic viability of the biorefinery, by representing contrasting approaches for the two principal residual streams generated during PLA production: for the xylose‐rich stream, succinic acid, and xylitol represent a higher‐value/higher‐processing‐complexity route and a lower‐complexity, commercially established route, respectively; for the lignin‐rich stream, adhesive production, and on‐site electricity generation represent a material‐oriented and an energy‐oriented valorization pathway, respectively. Comparing these options within a single framework allows the identification of the co‐product strategy most likely to offset production costs and improve overall economic feasibility, alongside its thermodynamic and environmental implications. Furthermore, previous studies have generally applied exergy, exergoeconomic, or environmental assessments independently, limiting understanding of how thermodynamic inefficiencies, cost formation mechanisms, and environmental burdens interact across integrated biorefinery systems. To address these gaps, this study develops and evaluates four integrated lignocellulosic biorefinery configurations for the co‐production of PLA and value‐added co‐products using corn stover and sugarcane bagasse as feedstocks. An integrated exergy, exergoeconomic, and exergoenvironmental framework is employed to quantify thermodynamic performance, identify cost and environmental burden formation mechanisms, and determine the key drivers influencing overall system sustainability.

The main contributions of this work are as follows. First, a comparative assessment of two major lignocellulosic feedstocks, corn stover and sugarcane bagasse, is conducted under identical biorefinery boundaries. Second, four alternative co‐product portfolios are systematically evaluated, encompassing both energy‐oriented, and material‐oriented lignin valorization pathways. Third, exergy, exergoeconomic, and exergoenvironmental analyses are integrated within a unified framework to jointly capture thermodynamic, economic, and environmental performance. Building on this framework, the study identifies whether feedstock selection or co‐product strategy exerts the dominant influence on overall system performance, and, in turn, develops design recommendations for sustainable PLA biorefineries based on the interactions between exergy destruction, cost formation, and environmental burden generation.

## 2 Materials and Methods

This study used SuperPro Designer v.13 and Excel to simulate a PLA biorefinery in four different scenarios, representing contrasting valorization strategies for the xylose‐rich stream (succinic acid vs. xylitol) and the lignin‐rich stream (electricity vs. lignin‐based adhesive) generated during PLA production (see Introduction for full rationale), to calculate mass and energy balance. The parameters suitable for the operating conditions and unit configurations simulated were obtained from literature and experimental data. A complete list of process parameters, feedstock compositions, conversion yields, operating conditions, economic assumptions, and material input inventories used in the simulations is provided in Table S6A,B. The processes described in the process flow diagrams (Figures S2–S5) were assumed to operates for 330 working days per year and for 7920 h per year (Figure 1). The plant capacity was determined to be 32 500 tonnes of lactic acid, reflecting the typical yearly output of existing lactic acid manufacturing facilities [23].

**FIGURE 1:**
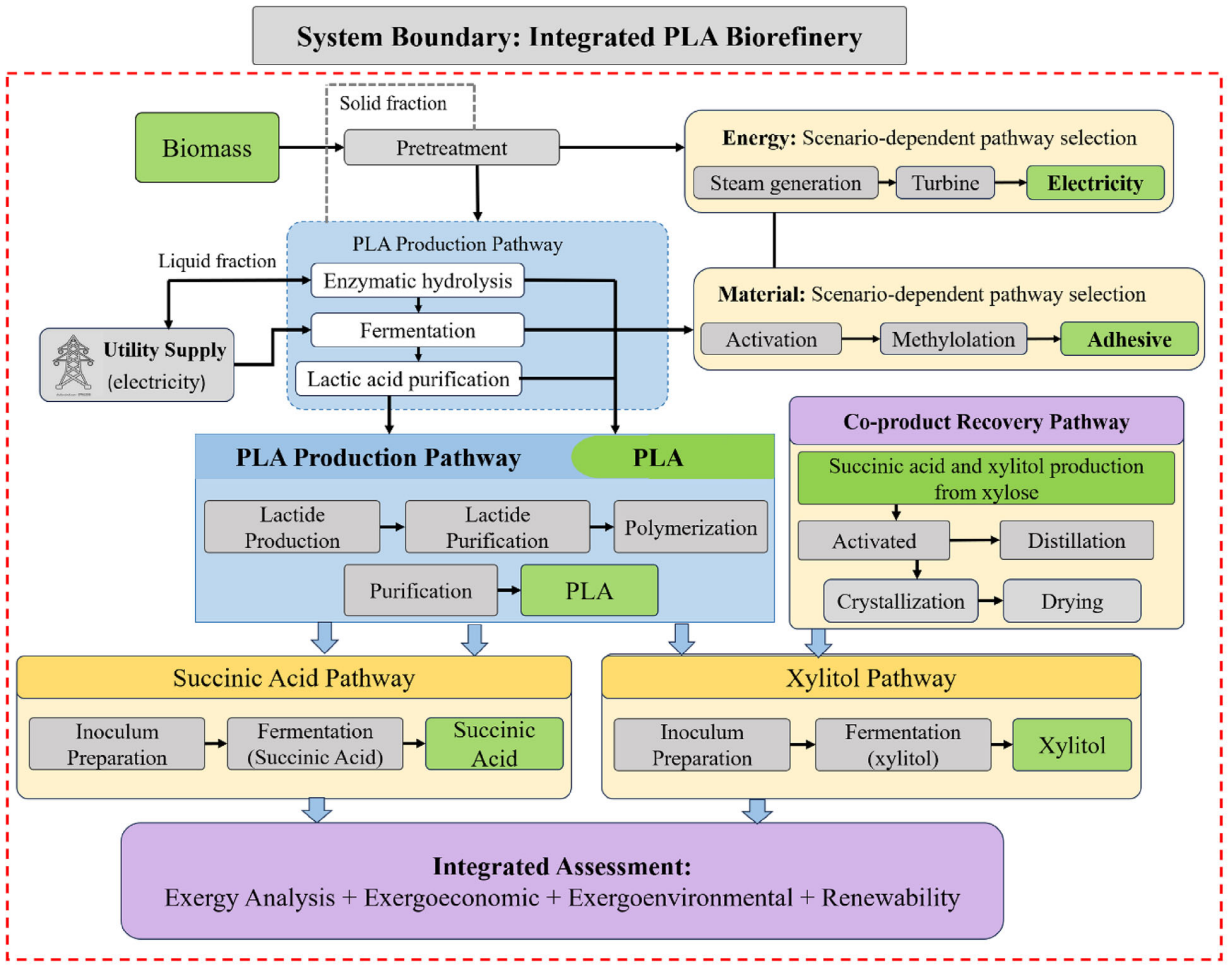
Diagram illustrating the four biorefinery pathways (Scenarios 1–4) designed for converting biomass into polylactic acid (PLA) and co‐products (succinic acid, xylitol, electricity, and lignin‐based adhesive). Each of the four pathways was applied independently to both corn stover and sugarcane bagasse feedstocks under identical process conditions, yielding the eight biorefinery configurations summarized in Table S1.

### 2.1 Process Description

In this study, to enable a rigorous comparative assessment, identical process configurations and operating conditions were applied to both feedstocks, thereby isolating the influence of intrinsic biomass composition on system performance. While feedstock‐specific optimization may improve absolute performance, it was beyond the scope of this study and would introduce confounding factors that hinder direct cross‐feedstock comparison. All unit operations, including alkaline pretreatment, enzymatic hydrolysis, fermentation, and downstream separation, were modelled using the same process parameters for both feedstocks. Accordingly, differences between the two systems arise exclusively from variations in intrinsic lignocellulosic composition rather than from modifications in process design or operating conditions. On a dry basis, corn stover contains 35% cellulose, 22% hemicellulose, and 16% lignin, along with 21.5% extractives and 5.5% ash, whereas sugarcane bagasse contains higher fractions of structural carbohydrates and lignin, comprising 43% cellulose, 31% hemicellulose, and 23% lignin, with lower extractives (1%) and ash (2%). These compositional differences influence biomass fractionation during alkaline pretreatment and subsequently affect fermentable sugar recovery, lignin distribution, utility demand, and downstream valorization pathways. Consequently, all observed differences in mass and energy flows, exergy behavior, and overall process performance are attributable to feedstock‐dependent compositional characteristics under equivalent processing conditions.

#### 2.1.1 PLA Production

Biomass undergoes alkaline pretreatment following Nalawade, Saikia [46], implemented as a continuous alkaline pretreatment process operated at 100°C, 20% solids, and a residence time of 120 min. After solid–liquid separation, the carbohydrate‑rich solids are subjected to enzymatic hydrolysis in stirred industrial reactors operated at 50°C, pH 5.2, and 20% (w/v) solids for 72 h, using Cellic CTec2 at an enzyme loading of 20 FPU g^−^^1^. The clarified hydrolysate is converted to lactic acid in fermentation units operated at 50°C and pH 7.0, supplemented with corn steep liquor and 60 g L^−^^1^ CaCO_3_. Fermentation is completed within 30 h, with full glucose consumption achieved after 24 h [46]. Product recovery and purification are achieved through integrated mechanical and ion‑exchange separation processes, including centrifugation, ultrafiltration, anion‑exchange adsorption (Amberlite IRA 96) with 4 N HCl elution, and final cation‑exchange polishing (Amberlite IR 120), yielding lactic acid with a purity of 99.2% [47, 48].

Lactide formation was modelled as a sequence of three industrial unit operations. First, lactic acid dehydration was carried out in a vacuum reactive evaporation step operated at 80°C and 60 kPa (abs) in the presence of 0.6 wt.% ZnO nanoparticles [49]. The dehydrated stream was subsequently transferred to a vacuum oligomerization reactor operated at 150°C and 10 kPa (abs) with a residence time of 3 h to promote oligomer formation [50]. The resulting oligomer was then subjected to thermal depolymerization at 220°C and 3 kPa (abs), with simultaneous vapor‑phase removal to enable lactide condenzation and rapid solidification [51]. Final purification was achieved through industrial recrystallization in ethyl acetate at a 1:1.5 (w/v) ratio, involving dissolution at 80°C followed by controlled cooling and solid–liquid separation [49, 52].

Ring‐opening polymerization (ROP) of L‑lactide to PLA is simulated in a jacketed polymerization reactor charged with 1 mol% Zn(Phe)_2_ catalyst in dry toluene and held under agitation for 12 h [53]. Polymer work‑up uses antisolvent precipitation by dispensing the polymer solution (in dichloromethane, DCM) into methanolic 5% HCl, followed by solid‐liquid separation and vacuum drying to constant mass [53]. Solvent management and recovery (DCM, toluene, and methanol) are incorporated through closed‑loop distillation.

#### 2.1.2 Xylose Valorization to Succinic Acid

During lactic acid production with Bacillus coagulans, xylose remains unconverted, and is diverted to succinic acid production. Following ion‑exchange conditioning, the xylose‑rich stream is concentrated by evaporation to 400 g L^−^^1^ [54]. The fermentation medium, excluding carbon sources, contains (g L^−^^1^): 5 yeast extract, 0.3 Na_2_HPO_4_, 1.4 NaH_2_PO_4_, 1 NaCl, 1.5 K_2_HPO_4_, 0.2 MgCl_2_·2H_2_O, and 0.2 CaCl_2_·2H_2_O, with MgCO_3_ supplied at 20 g L^−^^1^ as both a CO_2_ source and pH control agent. Production fermenters are inoculated at 10% (v/v) and operated at 37°C [54]. For inoculum preparation, Actinobacillus succinogenes DSM 22 257 is assumed to be maintained on TSA media containing (g L^−^^1^): 17 pancreatic digest of casein, 3 soy peptone, 2.5 glucose, 5 NaCl, 2.5 KH_2_PO_4_, and 18 agar, followed by expansion through a seed‑fermentation train operated at 37°C.

The fermentation broth is clarified by centrifugation to remove biomass, followed by activated‑carbon treatment for decolorization. The clarified solution is acidified to pH 2.0 and then concentrated by vacuum distillation at 60°C. Cooling crystallization at 4°C generates succinic acid crystals, which are recovered by filtration, optionally recentrifuged for washing, and dried at 70°C for 4 h to obtain purified product.

#### 2.1.3 Electricity Production

During pretreatment, the liquid fraction containing lignin‑rich liquor is separated. This lignin stream is allocated to on‑site energy recovery. Based on a heating value of 17.5 MJ kg^−^^1^ and assuming a 25% conversion efficiency, electricity generation was modelled. This system provides process electricity for pumps, agitation, and auxiliary systems. The allocation follows the approach defined in Nalawade et al. [46], using empirically derived lignin characteristics and heating values from the literature.

#### 2.1.4 Xylose Valorization to Xylitol

In the second scenario, the overall process configuration mirrors that of the primary pathway, with the distinction that the xylose‑rich stream is directed to xylitol production. Inoculum preparation is carried out in a seed fermenter using a medium containing (g L^−^^1^): 30 xylose, 40 rice bran extract, 2 (NH_4_)_2_SO_4_, and 1 CaCl_2_·2H_2_O, operated at 30°C with controlled agitation for 48 h, consistent with Thapa et al. [55]. Production‑scale fermentation employs the same nutrient formulation but excludes xylose, and fermenters are inoculated with 10% (v/v) seed culture. The fermentation step is operated at 30°C and 100 rpm with a residence time of 72 h (55). Xylitol recovery is initiated by biomass removal via centrifugation, followed by conditioning of the clarified stream with 15 g L^−^^1^ activated charcoal at 30°C for 1 h. The treated solution is then filtered and subjected to crystallization, washing, and drying operations to yield purified xylitol [55, 56].

#### 2.1.5 Lignin Valorization for Adhesive Production

In this scenario, lignin separated during alkaline pretreatment is routed to adhesive production. After pretreatment, the lignin‐rich liquor contains dissolved lignin alongside residual cellulose and hemicellulose. The stream first undergoes evaporation to remove excess water, followed by partial neutralization of residual sodium hydroxide using a 37% hydrochloric acid solution, reducing the NaOH‐to‐lignin mass ratio to the target level while retaining sufficient alkalinity to keep lignin in solution. Centrifugation is then used to remove residual solid cellulose and hemicellulose [57].

Lignin activation is performed at 80°C to form the activated lignin (AL) solution. Methylolation is then carried out at 60°C via dropwise addition of a 37% formaldehyde solution, achieving a methylolation reaction conversion of 80% to yield methylolated lignin (ML) [57].

The final lignin‐based phenol‐formaldehyde adhesive is synthesized by reacting the methylolated lignin with phenol and additional sodium hydroxide, achieving a phenol‐formaldehyde resin conversion of 90%. This synthesis stage was modelled with an exergy efficiency of 95.9% for both feedstocks [57].

### 2.2 Exergy Analysis

#### 2.2.1 Assumptions for Exergy Analysis

SuperPro simulations, together with Excel‐based mass balance calculations, were developed following the procedures outlinimnenerate the mass and energy flows for all process streams. The exergy analysis was then performed using the equations presented below and implemented under the following assumptions [58]:

1. The system is assumed to operate under steady‐state conditions. This assumption refers to the exergy‐accounting convention adopted for the analysis, rather than to the reaction kinetics of the underlying unit operations. Specifically, the exergy, exergoeconomic, and exergoenvironmental balances were evaluated using batch‐integrated inlet, and outlet exergy quantities, expressed over the complete batch cycle (kJ or GJ per batch), and subsequently normalized to the functional unit (1000 kg PLA); they were not evaluated as instantaneous, time‐dependent power (kW) flows. This convention does not imply that the underlying biochemical or polymerization reactions proceed under continuous, time‐invariant conditions: the batch and semi‐continuous residence times reported in Section 2.1 (e.g., 72 h enzymatic hydrolysis, 30 h lactic acid fermentation, 12 h PLA polymerization) reflect the process scheduling implemented in SuperPro Designer, consistent with the plant's annual operating basis of 32 500 t y^−^^1^ over 7920 h y^−^^1^ (330 working days).
2. Variations in kinetic and potential energy are considered insignificant.
3. The environmental reference state for all exergy calculations is defined as 298.15 K and 1.01325 bar for temperature and pressure, respectively.

#### 2.2.2 Exergy Analysis

The overall rates of energy transfer entering and leaving the steady‐state system are presented in Equation (1) [59].

(1) $$\dot{E}x_{\mathrm{system}}=\dot{E}x_{\mathrm{material}\;}+\dot{E}x_{Q}+\dot{E}x_{W}$$

Here, denotes the total exergy of the system (kW), while *Ėx_material_* corresponds to the exergy associated with the material mass flow (kW). The terms and represent the exergy contributions from heat transfer (kW) and work (kW), respectively. The values of and are obtained using Equations (2) and (3), where T_0_ and Ti are the reference temperature and temperature of the source, respectively [59]. Q̇ is the heat rate (kW) and Ẇ is power (kW).

(2) $$\dot{E}x_{Q}=\left(1-\frac{T_{0}\;}{Ti}\right)\;\dot{Q}$$

(3) $$\dot{E}x_{W}=\dot{W}$$

(4) $$\dot{E}x_{\mathrm{material}}=\dot{E}x_{\mathrm{ph}}+\dot{E}x_{\mathrm{ch}}+\dot{E}x_{\mathrm{potential}}+\dot{E}x_{\mathrm{kinetic}}$$

where *Ėx_ph_* denotes the physical exergy and *Ėx_ch_* the chemical exergy. *Ėx_potential_* and *Ėx_kinetic_* correspond to the potential and kinetic exergy terms, respectively [60]. As indicated in Equation (4), the physical exergy is determined from the mass flow rate $\dot{m}$ together with the specific enthalpy *h* and entropy *s* of the stream [61]. The subscript “0” refers to the properties at the restricted dead state, characterized by the reference pressure *P*_0_ and temperature *T*_0_.

(5) $$\dot{E}x_{ph}=\left[\left(h_{i}-h_{0}\right)-T_{0}\left(s_{i}-s_{0}\right)\right]$$

In this analysis, the specific enthalpy of each stream is obtained from the formulation provided in Equation (6) [62].

(6) $$h\;-\;h_{o}=C_{p}\;\left(T\;-\;To\right)$$

The specific entropy of each stream is determined using Equation (7) [63]

(7) $$S-S_{o}=C_{p}\ln\left(\frac{T}{T_{0}}\right)\;-R\ln\left(\frac{p}{P_{0}}\right)$$

In this formulation, *C_p_* corresponds to the specific heat capacity, *T *and *P* are the temperature and pressure of the stream, and *R* denotes the specific gas constant. The reference environment is characterised by a temperature of 298.15 K and a pressure of 1.01325 bar.

As reported by Banu et al. [61], determination of chemical exergy requires the mass fraction of each component, as expressed in Equation (8).

(8) $$\dot{E}x_{\mathrm{ch}}=\dot{n}\left[\sum y_{i}\varepsilon_{i}+R\;\cdot T_{0}\;\sum \;y_{i}\ln\left(y_{i}\right)\right]$$

Here, *n* refers to the molar flow rate (mol·s^−^^1^), *y_i_* is the mole fraction of species *i*, and ε_*i*_ denotes its standard chemical exergy (kJ·mol^−^^1^). The term *R* corresponds to the universal gas constant (kJ·mol^−^^1^·K^−^^1^), while *T*_0_ represents the reference temperature.

The specific chemical exergies of organic and inorganic species are available in the literature. For organic compounds, the chemical exergy can be approximated using Equation (9) [64].

(9) $$\begin{array}{ccc} ex_{\mathrm{ch}} & = & 363.439C+1075.633H\;-\;86.308O+4.14N\\ & & +190.798S\;-\;21.1A \end{array}$$

Here, C, H, O, N, S, and A denote the mass percentages of carbon, hydrogen, oxygen, nitrogen, sulfur, and ash in the organic component, respectively, and exch is expressed in kJ·kg^−^^1^. Equation (10) is then applied to convert the result from Equation (9) into the corresponding standard molar chemical exergy value (kJ·mol^−^^1^) [64, 65].

(10) $$\varepsilon =\frac{M\;.\;\;ex_{\mathrm{ch}}}{1000}$$

Here, ε denotes the standard molar chemical exergy of the component (kJ·mol^−^^1^), and represents the molar mass of the organic species (g·mol^−^^1^). When literature values are unavailable, the standard molar chemical exergy of inorganic components may be estimated using Equation (11).

(11) $$\varepsilon =-\Delta G+\sum_{P}n_{j}\varepsilon_{j}-\sum_{R}n_{k}\varepsilon_{k}$$

Here, G denotes the Gibbs free energy (kJ·mol^−^^1^), and n is the number of moles. Subscripts *P* and *R* correspond to products and reactants, respectively. Exergy destruction is then evaluated using Equation (12).

(12) $$\dot{E}x_{\mathrm{in}}\;-\;\dot{E}x_{\mathrm{out}}=\dot{E}x_{D}$$

Here, *Ėx_in_* is the inlet exergy (kW), *Ėx_out_* is the outlet exergy (kW), and *Ėx_D_* denotes the exergy destruction (kW).

#### 2.2.3 Exergy Efficiency

Exergetic efficiency is defined as the ratio of the useful (desired) output exergy to the input exergy [66]. In other words, a higher exergy efficiency reflects more effective utilization of high‐quality energy within the system [67]. The exergy efficiency (EE) is calculated using Equation (13) [68].

(13) $$\varphi =\frac{\mathrm{exergy}\;\mathrm{input}}{\mathrm{exergy}\;\mathrm{output}}=\frac{\sum \dot{E}x_{\mathrm{out}}}{\sum \dot{E}x_{\mathrm{in}}}\;\times 100=(1\;-\;\frac{\dot{E}x_{D}}{\dot{E}x_{\mathrm{in}}})\;\times 100$$

The exergy improvement potential (IP) rate (kW) is evaluated using Equation (14).

(14) $$IP=\left(1-\varphi \right)\left(\dot{E}x_{D}\right)$$

In Equation (14), the term (1 − φ) represents the fraction of a unit's exergy destruction that is considered avoidable and can, in principle, be recovered through improved design or operation, while the remaining fraction (φ) reflects exergy destruction retained at the unit's current operating efficiency, consistent with the approach adopted in comparable biorefinery exergy analyses [60].

(15) $$\varphi_{TOT}=\left(1-\frac{\dot{E}x_{D,TOT}}{\dot{E}x_{in,TOT}}\right)\;\times 100$$

The overall universal exergy efficiency, φ_TOT_, is calculated using Equation (15), where ${\dot{Ex}}_{D,\mathrm{TOT}}$ and ${\dot{Ex}}_{\mathrm{in},\mathrm{TOT}}$ denote the total exergy destruction and total exergy input, respectively.

### 2.3 Renewability Assessment

Berthiaume and Bouchard [69] advanced the cumulative exergy consumption (CExC) framework by introducing the concepts of cumulative net exergy consumption (CNEx) and restoration work (W_r_). The net exergy consumption, which indicates the total exergy losses accumulated throughout the process is defined as:

(16) $$CNEx=CExC\;-Ex_{p}\;$$

where Ex_p_ refers to chemical exergy of products.

Berthiaume et al. [70] introduced the notion of restoration work (Wr), which is determined by evaluating the cumulative net exergy consumption (CNEx) associated with non‑renewable resource inputs in both the production stage and the waste‑treatment stage. The restoration work required for depleting non‑renewable resources is given by the following equation:

(17) $$W_{r}={\text{CNEx}}_{p}+{\text{CNEx}}_{\mathrm{waste}}$$

In this formulation, CNEx_p_ and CNEx_waste_ refer to the cumulative net exergy consumption associated with resource processing and waste treatment, respectively. However, the CNEx related to waste treatment is not included in the calculation of restoration work.

Berthiaume et al. [70] introduced a renewability indicator derived from the concept of restoration work.

(18) $$\mathrm{RI}=\frac{W_{p}-\;W_{r}}{W_{p}}$$

where W_P_ refers to the produced work that was determined based on each scenario's final products. As a result, the renewability indicator (RI) equation is modified as follows:

(19) $$RI=\frac{Ex_{p}-\;W_{r}}{Ex_{p}}$$

Based on the value of RI, the following sections outline several scenarios that reflect varying degrees of renewability within the production process. The interpretation of the renewability indicator (RI) used in this study is illustrated in Figure 2.

**FIGURE 2:**
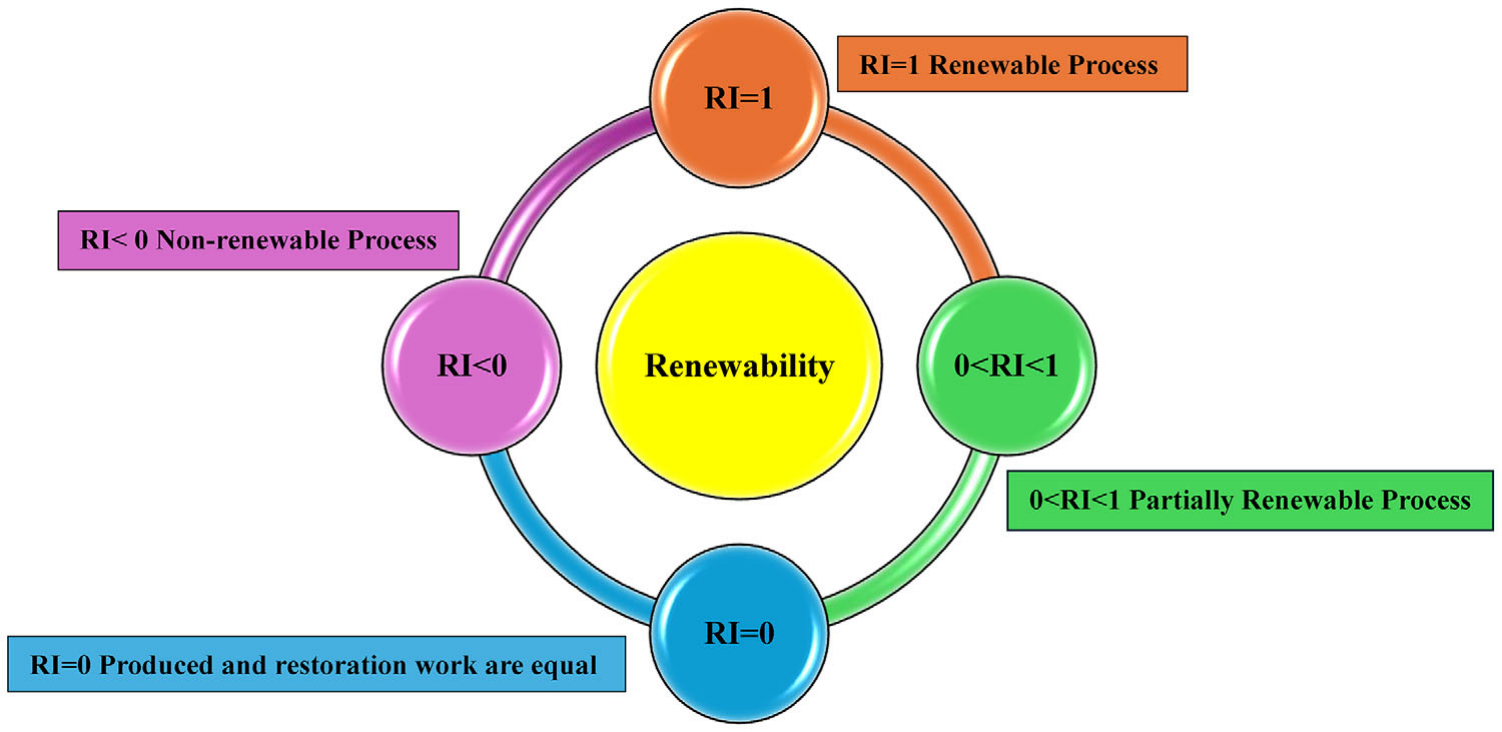
Renewability indicator (RI) definition.

### 2.4 Exergoenvironment Factor

Exergoenvironmental variables were employed to assess the environmental performance of individual process components. These variables were defined for each component following an approach analogous to the formulation of exergoeconomic variables in exergoeconomics [71]. In this study, the environmental burden was expressed in terms of kg CO_2_‐eq per batch, and the exergoenvironmental methodology was adapted accordingly. The specific environmental impact associated with the fuel of component *k* was calculated as (Equation 20).

(20) $$b_{F,K}=\frac{B_{F,K}}{E_{F,k}}$$

where is the specific environmental impact of the fuel of component *k*(kg CO_2_‐eq/GJ), *B*_*F*,*k*_ is the environmental burden associated with the fuel exergy of component *k*(kg CO_2_‐eq/batch), and *E*_*F*,*k*_ is the fuel exergy of the component (GJ/batch).

The environmental impact rate associated with the exergy destruction ${\dot{E}}_{D,k}$ occurring within the th component was determined as (25):

(21) $$B_{D,K}=b_{F,K}\times \;\;{\dot{E}}_{D,k}$$

where *B*_*D*,*k*_is the environmental impact of exergy destruction (kg CO_2_‐eq/batch), and *E*_*D*,*k*_ is the exergy destruction in component *k*(GJ/batch).

It should be noted that the environmental impact associated with exergy destruction in a given component should not be evaluated using the specific environmental impact of the fuel of the overall system. Instead, the specific environmental impact of the fuel of the th component must be used. Consequently, the relative position of a component within the system influences the environmental impact of exergy destruction, since depends directly on the local value of *b*_*F*,*k*_.

Within the exergoenvironmental framework, the total environmental burden associated with the th component was calculated by summing the environmental impact of exergy destruction and the component‐related environmental impact:

(22) $$\;B_{\mathrm{TOT},k}={\dot{\;Y}}_{k}+B_{D,k}$$

where *B*_*TOT*,*k*_is the total environmental burden of component *k*(kg CO_2_‐eq/batch), and *Y_k_* is the component‐related environmental impact (kg CO_2_‐eq/batch), which includes the environmental burdens associated with equipment construction, operation and maintenance, and disposal.

The exergoenvironmental factor was then used to evaluate the origin of the environmental burden within each component. It is defined as the ratio of the component‐related environmental impact to the total environmental burden of the component:

(23) $$f_{b,k}=\frac{{\dot{Y}}_{k}}{{\dot{Y}}_{k}+{\dot{B}}_{D,k}}=\frac{{\dot{Y}}_{k}}{{\dot{B}}_{TOT,k}}$$

where *f*_*b*,*k*_ is a dimensionless indicator.

A high value of *f*_*b*,*k*_ indicates that the total environmental burden of the component is dominated by component‐related impacts such as equipment manufacture, maintenance, and disposal. In contrast, a low value of indicates that the environmental burden is mainly caused by thermodynamic inefficiencies, i.e., exergy destruction within the component.

In general, when *f*_*b*,*k*_ > 0.7, the component‐related environmental impact *Y_k_* is considered the dominant contribution, whereas when *f*_*b*,*k*_ < 0.3, exergy destruction is considered the dominant source of environmental burden. Intermediate values indicate a mixed contribution from both sources

### 2.5 Exergoeconomic Analysis

For each unit operation, the fuel is defined as the total exergy entering the component, including the incoming process stream together with all external material and utility inputs (e.g., electricity, heat, steam, enzymes, chemicals, and water). The unit cost of the aggregated fuel stream is calculated as the exergy‐weighted average of all incoming streams, consistent with the Fuel rule of the SPECO (Specific Exergy Costing) methodology [71]. The product of each unit is defined as the useful exergy leaving the component and transferred to the subsequent process step; since each component in this study has a single aggregated product stream, the corresponding unit product cost is obtained directly from the exergoeconomic balance (Equation 24), which automatically satisfies the SPECO Product rule without requiring further allocation assumptions. Solvent and catalyst recovery within individual unit operations (e.g., toluene and Zn(Phe)_2_ during polymerization, and ethyl acetate during lactide purification) is represented through fixed recovery efficiencies (95%–99%), which determine the required make‐up quantity of each material; only these recovery‐adjusted make‐up quantities enter the fuel exergy and cost calculations. The same linear cost‐propagation principle is adopted for the exergoenvironmental analysis, in which the specific environmental impact of the fuel, bF,k, is determined from the exergy‐weighted average environmental impact of all incoming streams (Equation 20). The exergoeconomic balance for the process can be computed using the Equation (24):

(24) $$\sum C_{input,\;m}\dot{E}x_{input,\;m}+{\dot{Z}}_{tot}=\sum C_{output}\dot{E}x_{ouput,\;m}$$

Here, $\dot{C}$ denotes the cost rate, and ${\dot{Z}}_{\mathrm{tot}}$ represents the overall cost rate associated with plant construction and operation. The unit exergoeconomic cost is given by *C*, expressed as a cost rate (USD/h). The capital costs for all scenarios were estimated using SuperPro Economic Analyzer and are reported in Tables S7. The plant investment cost rate, $\dot{Z}$, is then calculated using Equation (25) [24].

(25) $$\dot{Z}=\frac{Z\;\cdot \;CRF\;\cdot \;\varphi_{m}}{H}$$

Here, *z* is the plant capital cost, *CRF* is the capital recovery factor, *φ* is the maintenance factor, and *H* denotes the annual operating hours. Capital costs obtained from SuperPro's equipment‐sizing routines were decomposed into installation, piping, instrumentation, and other cost components (Table S7) and combined with independently reconstructed fuel exergy costs (Table S9), rather than adopted directly from SuperPro's native costing outputs. The *CRF* is subsequently calculated using Equation (26).

(26) $$\mathrm{CRF}\;=\frac{i\;{\left(1+i\right)}^{N}}{{\left(1+I\right)}^{N}-1}$$

Here, N represents the reactor lifetime and (i) is the interest rate. The economic parameters employed in the exergoeconomic assessment of each biorefinery configuration are summarized in Table S6A. Following Amid et al. [24], the unit exergoeconomic cost of the fuel (c_f_) is defined in Equation (27).

(27) $$c_{f}=\frac{\sum_{m=1}C_{input,m}\dot{E}x_{input,m}}{\sum_{m=1}\dot{E}x_{input,m}}$$

The cost rate associated with exergy destruction in the process (${\dot{C}}_{D}$) is expressed by Equation (28).

(28) $${\dot{C}}_{D}=c_{f}\dot{E}x_{D}$$

The overall cost rate of the process is calculated using Equation (29).

(29) $${\dot{C}}_{tot}={\dot{Z}}_{tot}+c_{f}\dot{E}x_{D}$$

The exergoeconomic factor of the process (*f_c_*) is subsequently evaluated using Equation (30).

(30) $$f_{c}=\frac{{\dot{Z}}_{tot}}{Z_{tot}+c_{f}\dot{E}x_{D}}$$

The relative cost difference (*r_c_*) is then evaluated using Equation (31).

(31) $$r_{c}=\frac{c_{p}-c_{f}}{c_{f}}$$

## 3 Results and Discussion

### 3.1 Exergy Analysis

Exergy analysis offers a rigorous basis for evaluating the efficiency and sustainability of energy‑conversion pathways by quantifying where thermodynamic irreversibilities occur and how they propagate through the system. The thermophysical and chemical properties of all material streams across the evaluated scenarios are summarized in Table S6C, providing the foundational data required for subsequent exergy and cost‑based assessments.

#### 3.1.1 Exergy Destruction

Exergy‑destruction analysis was conducted to assess thermodynamic performance differences between corn stover, and sugarcane bagasse‐based PLA biorefineries across four co‑product configurations. Fuel exergy input was defined as the cumulative exergy of all material and utility streams entering the system, while product exergy comprised the exergy embodied in PLA and its co‑products. All results were normalized to a functional unit (FU) of 1000 kg PLA (GJ/FU). Figure 3 presents the spatial distribution of exergy inputs, useful outputs, and destruction across all process units for each scenario, illustrated for sugarcane bagasse‐based biorefineries in Figure 3a–d, with corresponding diagrams for corn stover provided in Section S11; link width is proportional to exergy magnitude, and red links denote exergy destroyed at each unit.

FIGURE 3Exergy flow (Sankey) diagrams tracking exergy inputs, useful outputs, and local irreversibility (exergy destroyed, shown in red) across all process units for sugarcane bagasse‐based PLA biorefineries: (a) Scenario 1, (b) Scenario 2, (c) Scenario 3, and (d) Scenario 4. Corresponding diagrams for corn stover‐based biorefineries, which exhibit a qualitatively similar pattern, are provided in Section S11.

**Figure:**
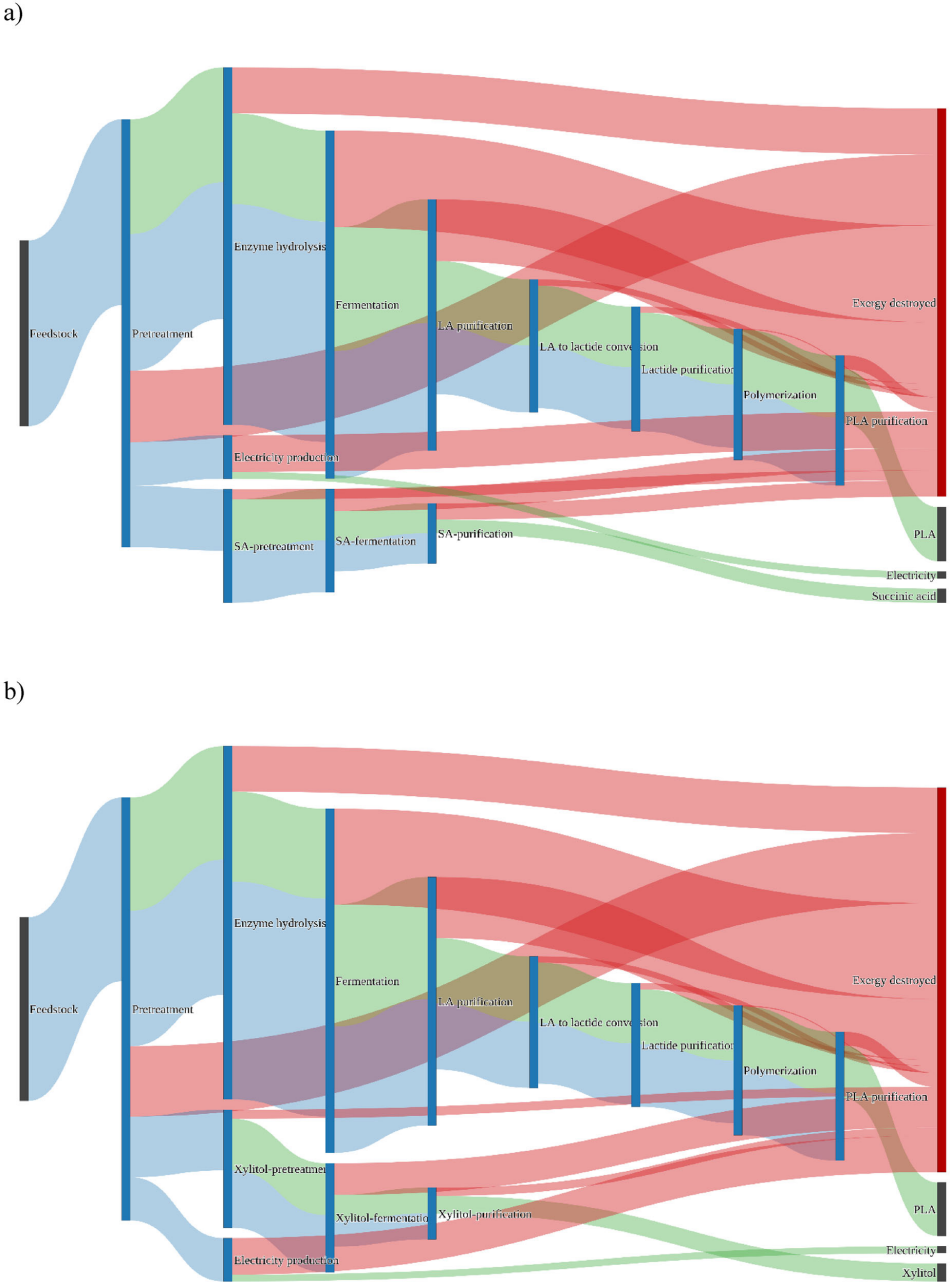

**Figure:**
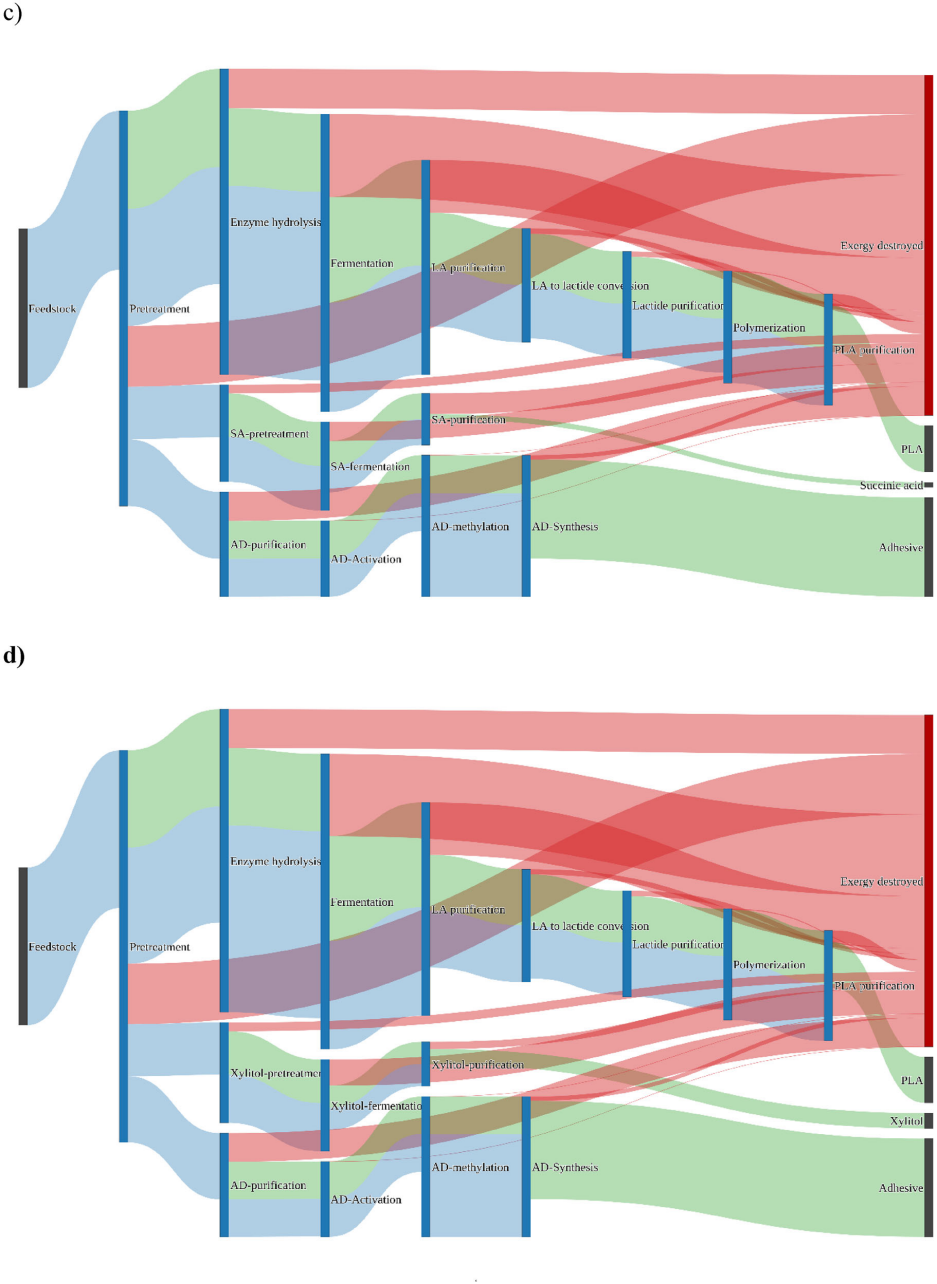

Across all configurations, fuel exergy input ranged from 97.41–128.49 GJ/FU for corn stover and 103.94–138.47 GJ/FU for sugarcane bagasse, with corresponding product exergy outputs of 44.04–81.41 GJ/FU and 49.12–90.75 GJ/FU, respectively (Table 1). These values yielded overall exergy efficiencies of 44%–64% for corn stover and 47%–65% for bagasse. In both feedstock systems, exergy destruction was primarily associated with co‐product recovery, and lignin‑valorization pathways, while the remaining irreversibilities were distributed between the core PLA production pathway and downstream separation operations.

**TABLE 1:** Exergy analysis results for four PLA biorefinery configurations: Overall exergy balance and stepwise exergy destruction by unit operation.

Parameter	Sc1		Sc2		Sc3		Sc4	
	Corn	Bagasse	Corn	Bagasse	Corn	Bagasse	Corn	Bagasse
**Overall Exergy Balance**								
Fuel exergy input (GJ/FU)	97.41	103.94	99.05	105.79	126.86	136.62	128.49	138.47
Product exergy output (GJ/FU)	47.71	49.12	44.04	50.64	81.41	89.22	77.74	90.75
Overall exergy destruction (GJ/FU)	49.70	54.81	55.01	55.14	45.45	47.39	50.75	47.72
Exergy efficiency (%)	48	47	44	47	64	65	60	65
**Stepwise Exergy Destruction by Unit Operation (GJ/FU)**								
Alkali pretreatment	1.47	1.20	1.47	1.20	1.47	1.20	1.47	1.20
Enzymatic hydrolysis	8.06	8.35	8.06	8.35	8.06	8.35	8.06	8.35
Lactic acid fermentation	0.57	2.22	0.57	2.22	0.57	2.22	0.57	2.22
Lactic acid purification	4.87	3.49	4.87	3.49	4.87	3.49	4.87	3.49
Lactic acid to lactide conversion	2.36	2.36	2.36	2.36	2.36	2.36	2.36	2.36
Lactide purification	2.29	2.33	2.29	2.33	2.29	2.33	2.29	2.33
PLA polymerization	0.36	0.37	0.36	0.37	0.36	0.37	0.36	0.37
PLA purification	2.37	2.39	2.37	2.39	2.37	2.39	2.37	2.39
Electricity generation	11.78	13.88	11.78	13.88	—	—	—	—
SA pretreatment	3.34	3.83	—		3.34	3.83	—	
SA fermentation	8.23	8.30	—		8.23	8.30	—	
SA purification	3.94	6.09	—		3.94	6.09	—	
Xylitol pretreatment	—	—	5.02	3.40	—	—	5.02	3.40
Xylitol fermentation	—	—	10.45	12.04	—	—	10.45	12.04
Xylitol purification			5.35	3.12		5.35	3.12	
Lignin adhesive block (total)	—	—	—		7.53	6.45	7.53	6.45

In electricity‑integrated configurations (Scenarios 1 and 2), total exergy destruction for corn stover ranged from 49.70–55.01 GJ/FU, while sugarcane bagasse exhibited values between 54.81–55.14 GJ/FU (Table 1). Electricity generation represented the single largest source of irreversibility in these scenarios, accounting for 11.78 GJ/FU in corn stover systems, and 13.88 GJ/FU in bagasse‑based systems. These contributions correspond to approximately 22%–27% of total exergy destruction, confirming the substantial thermodynamic penalty associated with combustion‑based energy recovery. Relative to corn stover, the higher electricity‐related exergy destruction in bagasse‐based systems arises from its greater lignin content, which increases the amount of lignin directed to combustion and thereby amplifies irreversibilities associated with energy conversion.

When succinic acid was co‑produced (Scenario 1), both feedstocks displayed similar exergy‑destruction structures. Succinic‑acid pretreatment, fermentation, and purification together constituted the dominant thermodynamic hotspot, contributing approximately 15.5 GJ/FU for corn stover, and 18.2 GJ/FU for sugarcane bagasse (Table 1). Enzymatic hydrolysis represented the next largest contributor in both systems (8.06–8.35 GJ/FU). By comparison, alkali pretreatment and PLA polymerization each accounted for less than 5% of total exergy destruction. Bagasse‐based configurations exhibit higher destruction during lactic‐acid fermentation and succinic‐acid purification due to their higher cellulose and hemicellulose content, which increases the generation of fermentable sugars and subsequently intensifies downstream processing loads. This results in greater separation demand and higher solid‐liquid handling requirements, thereby amplifying irreversibilities in fermentation and purification units.

Substitution of succinic acid with xylitol (Scenario 2) resulted in the highest overall exergy destruction among all configurations for both feedstocks. Xylitol fermentation emerged as the principal thermodynamic hotspot, contributing 10.45 GJ/FU for corn stover and 12.04 GJ/FU for sugarcane bagasse, with additional losses arising from pretreatment and purification (Table 1). Consequently, Scenario 2 exhibited the lowest overall exergy efficiencies across all configurations (44% for corn stover and 47% for bagasse), indicating that sugar‑alcohol recovery imposes a substantial thermodynamic penalty irrespective of feedstock choice.

A pronounced reduction in total exergy destruction was observed when lignin was valorized into adhesive products instead of electricity (Scenarios 3 and 4). For corn stover, overall exergy destruction decreased to 45.45 GJ/FU in Scenario 3 and 50.75 GJ/FU in Scenario 4, while sugarcane bagasse exhibited corresponding values of 47.39 GJ/FU and 47.72 GJ/FU (Table 1). In these configurations, electricity‑related irreversibilities were eliminated and replaced by destruction within the lignin‑adhesive block, which remained lower in magnitude (6.45–7.53 GJ/FU) than the electricity pathway. Scenario 3 achieved the highest exergy efficiencies for both feedstocks, demonstrating the thermodynamic advantage of material‑based lignin valorization over energy recovery.

Across all scenarios, the core PLA production pathway comprising alkali pretreatment, enzymatic hydrolysis, lactic‑acid fermentation, lactide synthesis, and PLA polymerization operated as a comparatively exergy‑efficient subsystem relative to co‑product recovery and energy‑generation routes. Within this pathway, enzymatic hydrolysis and lactic‑acid purification were the dominant contributors (approximately 8–11 GJ/FU combined), while lactide conversion and polymerization contributed less than 3 GJ/FU each (Table 1). The similarity in PLA‑train performance between corn stover and sugarcane bagasse indicates that differences in system‑level exergy destruction arise primarily from co‑product selection and lignin‑valorization strategy rather than from inefficiencies in PLA synthesis itself.

Overall, corn stover and sugarcane bagasse exhibited qualitatively similar exergy‑destruction patterns across all four biorefinery configurations. Quantitative differences arose from feedstock‑dependent impacts on energy recovery and separation intensity, with sugarcane bagasse generally exhibiting higher irreversibility in electricity‑coupled systems and xylitol fermentation. Among all configurations, the adhesive‑integrated Scenario 3 delivered the most favorable thermodynamic performance, whereas the xylitol‑based Scenario 2 consistently resulted in the highest exergy destruction. These results demonstrate that co‑product pathway selection exerts a stronger influence on system‑level exergy destruction than feedstock choice alone in lignocellulosic PLA biorefineries.

#### 3.1.2 Exergy Improvement Potential Rate and Universal Exergy Efficiency

Exergy improvement potential analysis was performed to identify process units that contribute most significantly to thermodynamic irreversibilities and therefore represent priority targets for optimization in corn stover and sugarcane bagasse‐based PLA biorefineries. The analysis complements exergy‑destruction results by quantifying the recoverable fraction of destroyed exergy and highlighting the relative importance of individual sub‑systems across alternative co‑product configurations. A complete ranking of unit operations by improvement potential (IP), together with technically feasible optimization strategies for each identified hotspot, is provided for both feedstocks and all four scenarios in Table S10.

##### 3.1.2.1 Exergy Improvement Potential

For corn stover‐based systems, Scenario 1 exhibits a total improvement potential of 18.22 GJ/FU. The largest contribution originates from electricity generation, accounting for 54% of the total improvement potential, followed by succinic acid (SA) fermentation (20%), SA purification (8%), and enzymatic hydrolysis (7%). Together, electricity generation and SA fermentation account for more than two‐thirds of the total improvement potential, indicating these units as the primary contributors to thermodynamic irreversibility. In Scenario 2, the total improvement potential for corn stover increases to 22.05 GJ/FU, driven primarily by electricity generation (45%), and xylitol fermentation (25%). Secondary contributions arise from xylitol purification (14%) and enzymatic hydrolysis (6%). Improvement potential in this configuration is therefore mainly associated with the power‐generation block and xylitol fermentation, with additional contributions from hydrolysis and downstream purification. Scenario 3 shows a substantially lower total improvement potential of 9.61 GJ/FU for corn stover, reflecting improved overall thermodynamic performance. Improvement potential is concentrated in SA fermentation (39%) and SA purification (16%), with further contributions from enzymatic hydrolysis (14%) and adhesive (AD) purification (13%). In Scenario 4, the improvement potential for corn stover increases to 13.44 GJ/FU. Xylitol fermentation again represents the dominant contributor (41%), followed by xylitol purification (22%), enzymatic hydrolysis (10%), and AD purification (9%). This distribution of improvement potential is consistent with previous exergy studies, in which fermentation units were identified as major sources of irreversibility due to highly irreversible biochemical reactions and associated heat losses, while downstream recovery processes involve additional energy demand and rapid heat and mass transfer [59].

Electricity generation was the single largest exergy‐destroying unit across all configurations (11.78–13.88 GJ/FU), despite converting lignin combustion into a comparatively low‐exergy product (electricity). This reflects a fundamental thermodynamic limitation of combustion‐based power generation: converting the high‐quality chemical exergy of lignin into heat via combustion is inherently irreversible, and the subsequent heat‐to‐work conversion through the steam‐turbine cycle is further bounded by Carnot efficiency. In effect, two irreversible conversions are stacked, chemical‐to‐thermal and thermal‐to‐mechanical, each discarding useful exergy. This explains why the adhesive‐integrated configurations (Scenarios 3 and 4), which instead retain lignin's chemical structure for a material product, reduce total exergy destruction relative to the electricity‐integrated scenarios: material valorization avoids the double thermodynamic penalty of combustion entirely. Where electricity generation from lignin is still required, practical remedies include integrating the combustion step into a combined heat and power (CHP) arrangement so that the Carnot‐rejected heat is captured for process heating rather than discarded, and specifying higher‐efficiency turbine/generator equipment to reduce the mechanical conversion penalty.

Xylitol fermentation was the second‐largest hotspot (10.45–12.04 GJ/FU), exceeding even the succinic acid fermentation step (8.23–8.30 GJ/FU) despite operating on the same xylose‐rich stream. This is consistent with the substantially longer residence time required for xylitol fermentation relative to succinic acid fermentation, reflecting the comparatively slower bioconversion kinetics of microbial xylitol production, which increases the cumulative utility and physical exergy input per unit of product recovered. Practical remedies include adopting fermentation strategies that improve volumetric productivity (e.g., higher cell‐density operation or process intensification) to reduce residence time, and evaluating alternative xylose‐fermenting strains with higher productivity, which would proportionally reduce the utility exergy demand associated with this step.

Enzymatic hydrolysis remained a consistent hotspot across all four co‐product scenarios (8.06 GJ/FU for corn stover, 8.35 GJ/FU for bagasse), since it forms part of the core PLA production pathway shared by every configuration. This destruction arises primarily from the combination of enzyme protein input (itself embodying non‐trivial chemical exergy) and the long residence time (72 h) required at moderate solids loading (20% w/v) to achieve adequate glucan conversion. Remedies include enzyme recycling or immobilization to reduce the net enzyme exergy input per batch, and adoption of simultaneous saccharification and fermentation (SSF) configurations, which would eliminate a discrete hydrolysis‐to‐fermentation transfer step and its associated equipment and thermal losses.

For sugarcane bagasse‐based systems, Scenario 1 exhibits a total improvement potential of 21.51 GJ/FU. Electricity generation represents the largest contribution (54%), followed by SA fermentation (16%), SA purification (15%), and enzymatic hydrolysis (6%). Electricity generation and SA fermentation together account for more than two‐thirds of the total improvement potential. In Scenario 2, improvement potential for bagasse is dominated by electricity generation (53%) and xylitol fermentation (30%), with smaller contributions from enzymatic hydrolysis (6%) and xylitol purification (4%). Scenario 3 shows improvement potential concentrated in SA fermentation (33%) and SA purification (31%), with additional contributions from enzymatic hydrolysis (13%) and AD purification (7%). In Scenario 4, xylitol fermentation dominates improvement potential (59%), followed by enzymatic hydrolysis (12%), xylitol purification (9%), and AD purification (6%). Overall, improvement potential analysis for both feedstocks indicates that recoverable irreversibilities are primarily concentrated in co‐product recovery processes and electricity generation, whereas the core PLA production pathway contributes comparatively less.

##### 3.1.2.2 Universal Exergy Efficiency

Universal exergy efficiency analysis quantifies the intrinsic thermodynamic performance of individual process units, as illustrated in Figure 4e,f. For both feedstocks, the baseline PLA production pathway operates with consistently high efficiency across all scenarios, with values ranging from 83.80% to 98.46%. Alkali pretreatment (98%), polymerization (98%), and fermentation (95%) exhibit particularly high efficiencies. Lactic‐acid purification also maintains high efficiency (≈93%), while lactide conversion and purification achieve efficiencies close to or exceeding 90%. Enzymatic hydrolysis exhibits intermediate universal exergy efficiency (84%). PLA purification achieves efficiencies around 90.7%, indicating moderate exergy losses during final separation. In contrast, SA and xylitol co‐product pathways exhibit lower and more variable efficiencies. Pretreatment steps typically operate at 83%–85% efficiency, while fermentation efficiencies range from approximately 44.8% to 57.6%. Purification stages display the widest variability (46.4%–68.5%), consistent with the energy‐intensive nature of separation processes. Electricity generation remains the least efficient sub‐unit overall, with a universal exergy efficiency of approximately 15.8%. In adhesive‐integrated scenarios (Scenarios 3 and 4), the additional lignin‐valorization steps operate at high efficiencies, including activation (99.8%), methylolation (99.8%), synthesis termination (95.9%), and adhesive purification (84%). Adhesive integration does not introduce significant inefficiencies at the unit‐operation level, and lower efficiencies remain concentrated in co‐product processing and electricity generation. Overall, the baseline PLA production pathway exhibits consistently high universal exergy efficiency across all scenarios. Lower efficiencies are primarily associated with co‐product recovery and energy‐conversion steps, which dominate system‐level thermodynamic limitations in both corn stover and sugarcane bagasse–based PLA biorefineries.

FIGURE 4Distribution of exergy improvement potential among process areas for (a) Scenario 1, (b) Scenario 2, (c) Scenario 3, and (d) Scenario 4, and universal exergy efficiency (%) of individual process units across all four scenarios for (e) sugarcane bagasse and (f) corn stover‐based PLA biorefineries.

**Figure:**
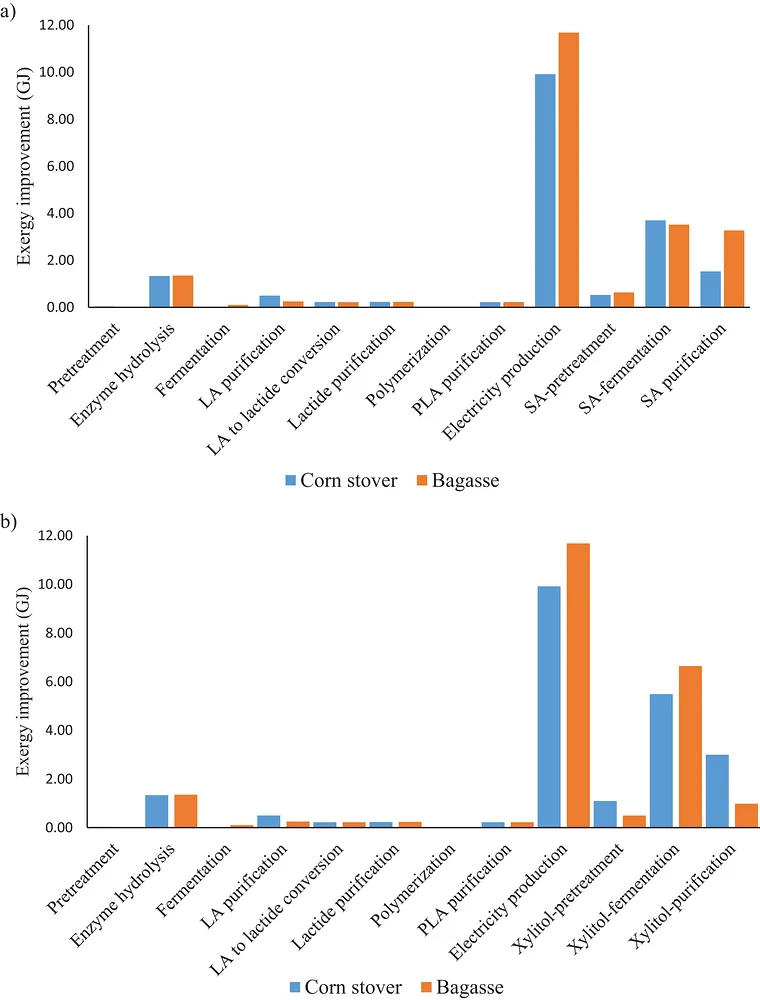

**Figure:**
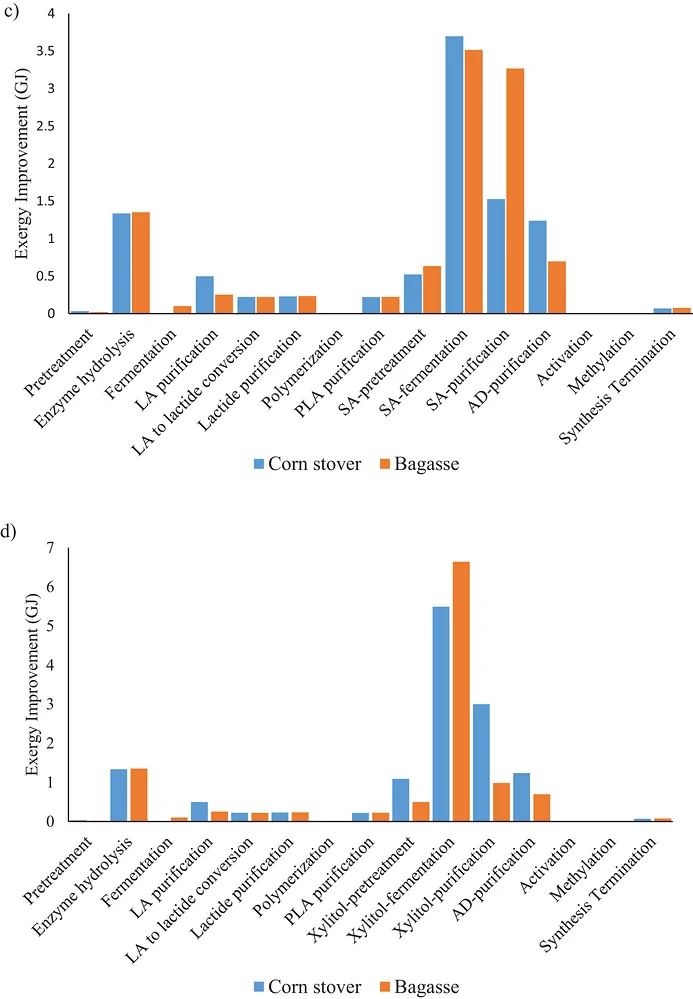

**Figure:**
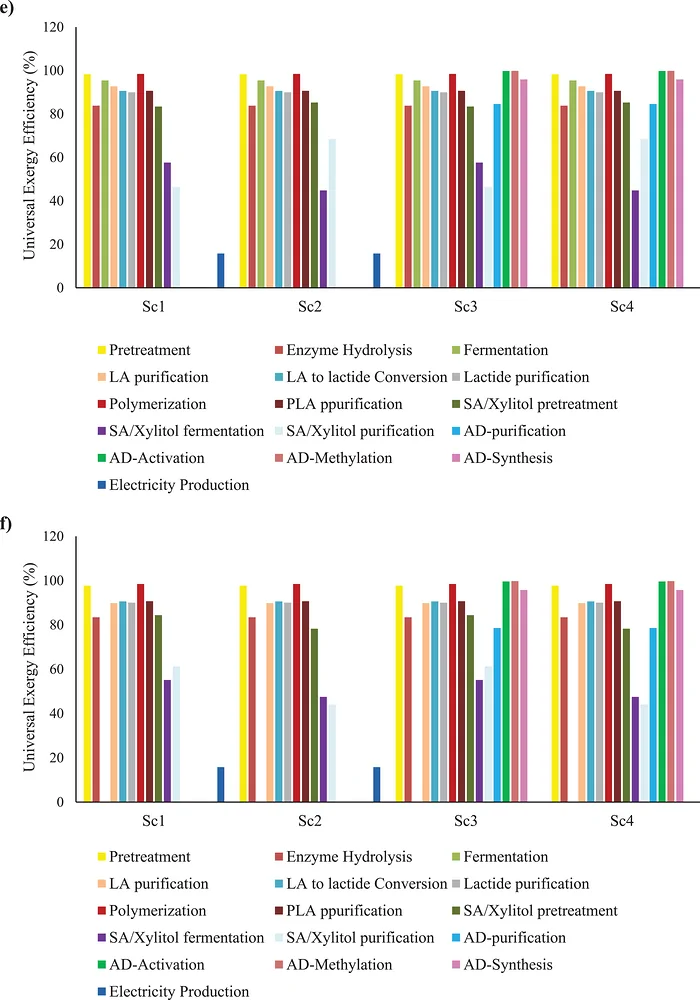

#### 3.1.3 Renewability Indicator (RI)

The renewability indicator (RI), calculated following the method proposed by Berthiaume, Bouchard and Rosen (70), was employed to further assess the thermodynamic sustainability of the evaluated biorefinery configurations. RI provides an integrated measure of the balance between useful product exergy and the cumulative exergy consumed from non‐renewable resources, thereby enabling classification of each configuration as non‐renewable (RI < 0), partially renewable (0 < RI < 1), or fully renewable (RI  =  1).

For the corn stover–based biorefinery, Scenarios 3 and 4, which incorporate lignin valorization to adhesive, yield positive RI values of 0.442 and 0.347, respectively, classifying both configurations as partially renewable. In contrast, Scenarios 1 and 2, where lignin is directed to electricity generation, exhibit negative RI values of −0.042 and −0.249, indicating non‐renewable behavior. Notably, Scenario 1 approaches thermodynamic neutrality (RI ≈ 0), reflecting relatively high recovery of useful product exergy through succinic‐acid co‐production despite the substantial irreversibilities associated with combustion‐based electricity generation. These trends are fully consistent with the universal exergy efficiency (44%–64%), functional exergy efficiency (27%–48%), and normalized exergy destruction (0.56–1.25 GJ GJ^−^^1^ product) results for corn stover, collectively confirming that lignin valorization to adhesive rather than electricity is the primary determinant of thermodynamic renewability.

A similar but quantitatively distinct pattern is observed for the sugarcane bagasse‐based biorefinery. Scenarios 1 and 2 yield negative RI values of −0.116 and −0.089, respectively, confirming their classification as non‐renewable when lignin is diverted to electricity production. In contrast, adhesive‐integrated Scenarios 3 and 4 achieve positive RI values of 0.469 and 0.474, indicating partially renewable system performance. Compared with corn stover, the bagasse‐based configurations exhibit slightly higher RI values in adhesive‐integrated scenarios, which can be attributed to the higher lignin content of bagasse that increases the fraction of biomass directed toward material‐based valorization rather than combustion, thereby enhancing the conversion of feedstock exergy into useful products and reducing the relative contribution of non‐renewable exergy inputs. The similar magnitude of RI values for Scenarios 3 and 4 further indicates that the thermodynamic benefit of adhesive integration dominates over differences arising from the choice between succinic acid and xylitol as co‐products, as lignin valorization governs the primary exergy conversion pathway while the sugar‐derived co‐product routes contribute comparatively less to overall exergy distribution.

Across both feedstocks, the renewability analysis consistently demonstrates that electricity‐coupled configurations remain thermodynamically non‐renewable under the examined process assumptions, whereas material‐based lignin valorization enables partial renewability. These findings reinforce the conclusions drawn from the exergy and exergoeconomic analyses, highlighting that the selection of the lignin valorization pathway exerts a stronger influence on system‐level sustainability than feedstock choice alone. Overall, adhesive‐integrated Scenarios 3 and 4 represent the most favorable pathways from a renewability perspective for both corn stover and sugarcane bagasse biorefineries, further supporting material‐oriented lignin valorization as a key strategy for improving the thermodynamic sustainability of integrated PLA biorefineries.

### 3.2 Exergoenvironmental Analysis

Exergoenvironmental analysis was conducted to quantify the environmental burdens associated with thermodynamic irreversibilities and to distinguish whether these burdens originate primarily from exergy destruction or from component‐related impacts. Figure 5 illustrates the distribution of the exergoenvironmental factor and the environmental impact of exergy destruction for all system components.

FIGURE 5Distribution of the exergoenvironmental factor and the environmental impact associated with exergy destruction for the system components for corn stover (a–d) and bagasse (e–h).

**Figure:**
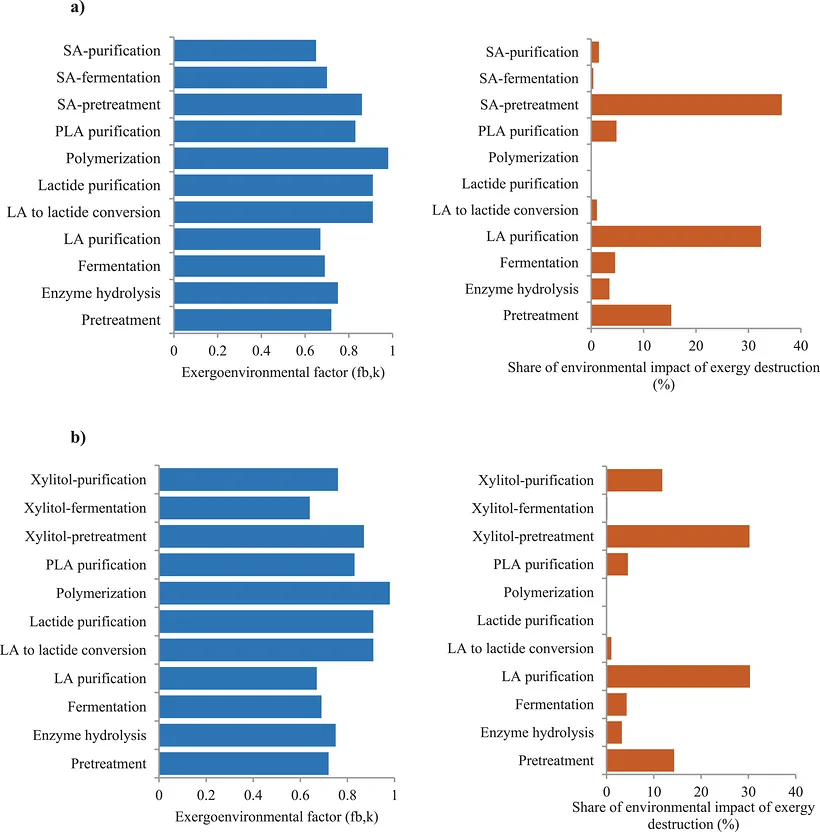

**Figure:**
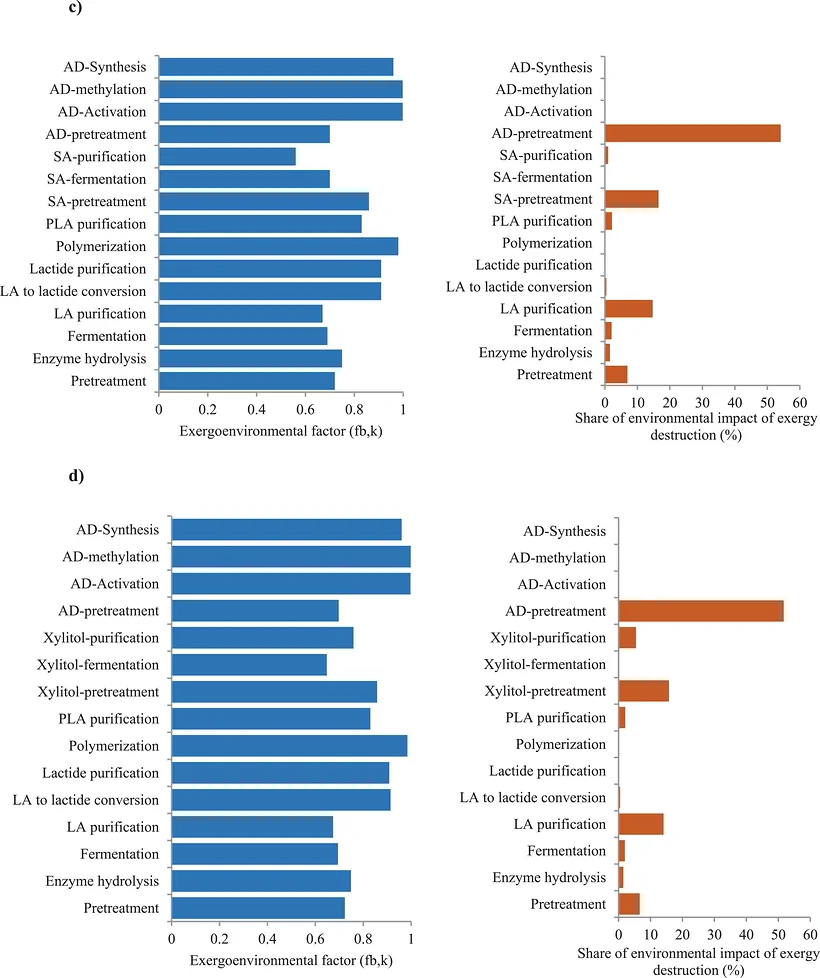

**Figure:**
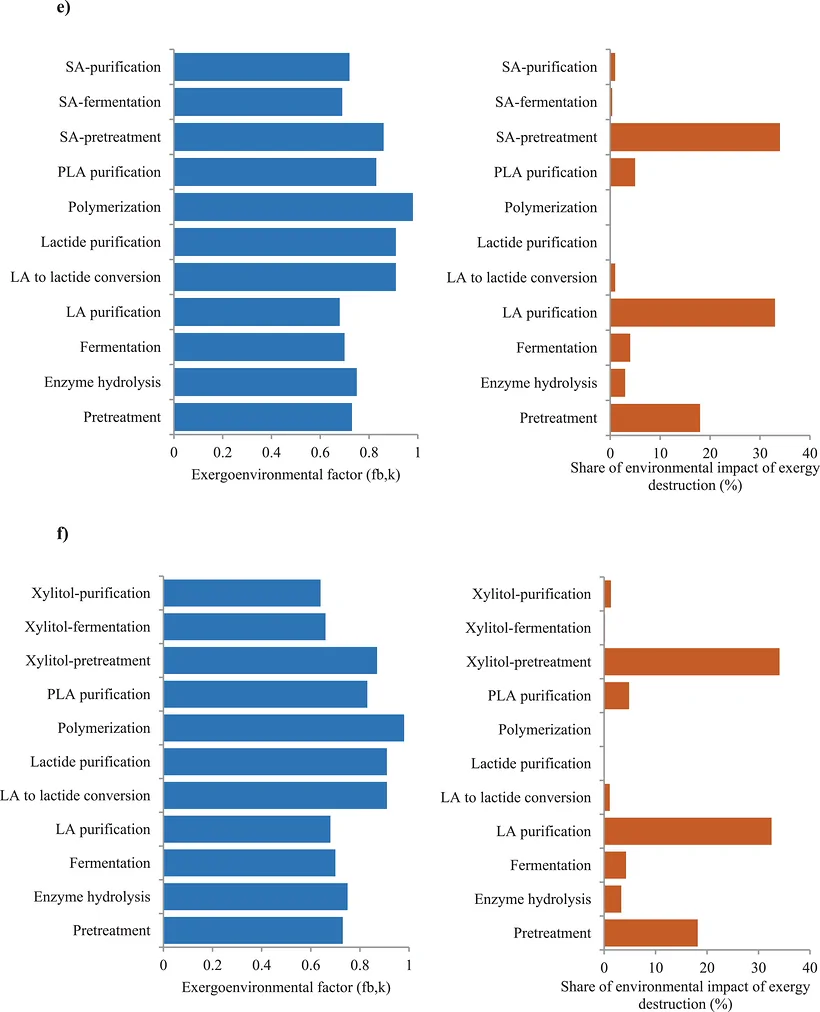

**Figure:**
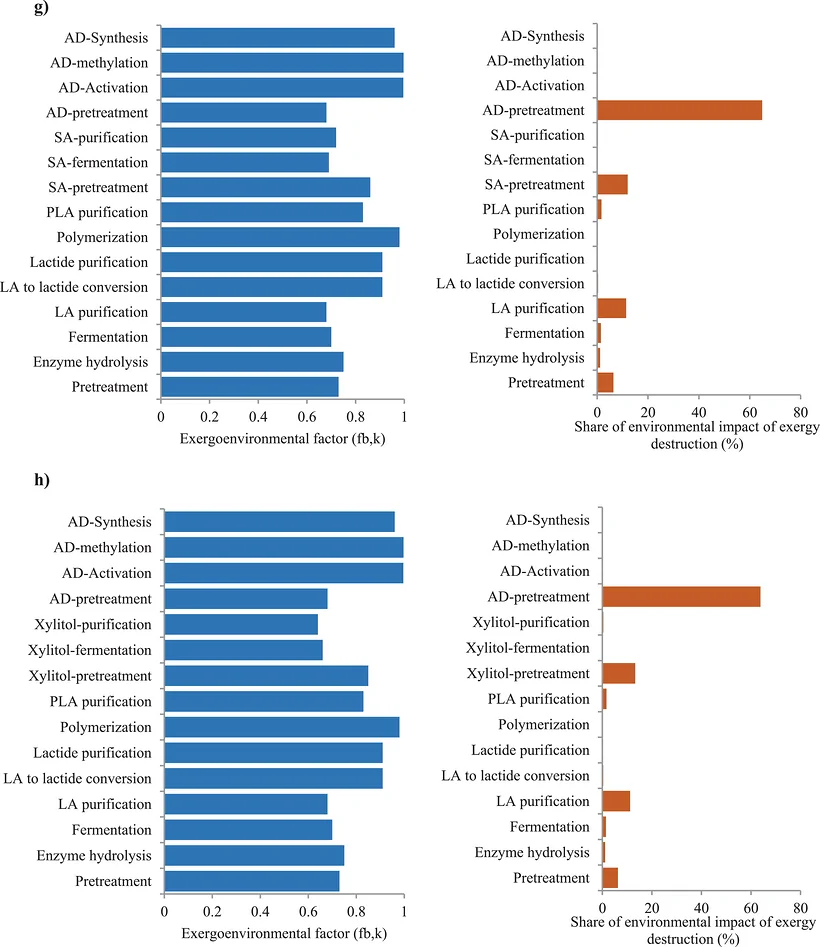

For corn stover‐based Scenario 1 (Figure 5a), pretreatment, enzymatic hydrolysis, and fermentation exhibit moderate exergoenvironmental factors (*f*_*b*,*k*_ = 0.70‐0.75)), reflecting comparable contributions from component‐related impacts and thermodynamic inefficiencies. Pretreatment and fermentation generate environmental impacts of exergy destruction of 34 485 and 8059 kg CO_2_‐eq batch^−^^1^, respectively. In contrast, lactic‐acid purification represents the dominant environmental hotspot, with a high impact of exergy destruction (61 700 kg CO_2_‐eq batch^−^^1^) and a total environmental burden of 189 870 kg CO_2_‐eq batch^−^^1^, confirming the strong contribution of separation‐driven irreversibilities. Polymerization and lactide purification exhibit high exergoenvironmental factors (*f*_*b*,*k*_ > 0.90)), demonstrating that their environmental burdens are primarily associated with component‐related impacts.

Within the succinic‐acid pathway, SA pretreatment shows the largest total environmental burden of all units (480 491 kg CO_2_‐eq batch^−^^1^), with a high exergoenvironmental factor (0.865), confirming the dominance of construction‐ and material‐related impacts over exergy destruction. In contrast, SA fermentation and SA purification display lower exergoenvironmental factors (0.69–0.72), indicating a greater contribution from thermodynamic inefficiencies.

In Scenario 2 (Figure 5b), replacement of succinic acid with xylitol intensifies environmental burdens in the co‐product train. Xylitol pretreatment remains the dominant contributor, with a total environmental burden of 480 163 kg CO_2_‐eq batch^−^^1^ and an exergoenvironmental factor of 0.865. Xylitol fermentation and purification exhibit lower exergoenvironmental factors (0.64–0.66), demonstrating stronger coupling between exergy destruction and environmental impact.

Scenario 3 (Figure 5c), which integrates lignin valorization to adhesive while retaining succinic acid, shows a redistribution of environmental burdens. Electricity‐related impacts are eliminated; however, AD pretreatment becomes the dominant contributor, exhibiting the highest total environmental burden in this scenario (1085 428 kg CO_2_‐eq batch^−^^1^). Adhesive activation, methylolation, and synthesis show very high exergoenvironmental factors (0.96–0.998), demonstrating that their environmental burdens are almost entirely associated with component‐related impacts rather than thermodynamic irreversibilities.

Scenario 4 (Figure 5d), combining xylitol production with adhesive integration, exhibits a similar pattern. Xylitol pretreatment contributes approximately 489 000 kg CO_2_‐eq batch^−^^1^, while AD pretreatment again dominates the total environmental burden (1.09 × 10^6^ kg CO_2_‐eq batch^−^^1^). As in Scenario 3, adhesive upgrading steps are characterized by very high exergoenvironmental factors, indicating a limited contribution from thermodynamic irreversibility.

Bagasse‐based scenarios (Figure 5e–h) exhibit trends consistent with corn stover, with slightly higher environmental burdens due to increased specific environmental impacts of supplied utilities. Lactic‐acid purification remains the dominant contributor to the environmental impact of exergy destruction (≈ 62 300 kg CO_2_‐eq batch^−^^1^), while xylitol purification exhibits high total environmental burdens on the order of 1.0 × 10^5^ kg CO_2_‐eq batch^−^^1^. Adhesive‐integrated configurations again show very high exergoenvironmental factors (> 0.95) for activation, methylolation, and synthesis, confirming the predominance of component‐related environmental impacts.

Overall, exergoenvironmental analysis shows that environmental burdens in the examined biorefineries are primarily associated with separation‐intensive purification processes and co‐product pretreatment stages, while PLA polymerization and adhesive upgrading units exhibit limited irreversibility‐driven impacts. Scenarios incorporating lignin valorization to material products rather than electricity exhibit lower exergy‐destruction‐related environmental impacts, with Scenario 3 exhibiting the most balanced distribution of environmental burdens among system components.

These findings point to two mechanistically distinct types of environmental hotspot. Pretreatment stages within the succinic acid and xylitol pathways carry the largest total environmental burdens (480 491 and 480 163 kg CO_2_‐eq batch^−^^1^, respectively), yet their high exergoenvironmental factors (0.865) indicate that this burden originates predominantly from the embodied environmental impact of the chemicals consumed during conditioning, rather than from thermodynamic inefficiency. This contrasts with lactic acid purification, which represents the dominant environmental hotspot within the core PLA pathway (61 700 kg CO_2_‐eq batch^−^^1^ destruction impact; 189 870 kg CO_2_‐eq batch^−^^1^ total burden), reflecting a stronger relative contribution from separation‐related irreversibility than the pretreatment units discussed above. This distinction has direct practical implications: mitigating pretreatment burdens is likely to require reagent substitution and recovery, adopting lower‐impact conditioning reagents, increasing regenerant recycling rates, and reducing net chemical dosing through process intensification, whereas reducing the burden associated with lactic acid purification would benefit more directly from measures that lower separation‐related exergy destruction, such as those discussed in Section 3.2.4.

The adhesive‐integrated scenarios illustrate the component‐driven pattern even more strongly. Eliminating electricity generation removes its associated environmental burden entirely, yet AD pretreatment emerges as the new dominant contributor in Scenarios 3 and 4, reaching total environmental burdens of 1085 428 kg CO_2_‐eq batch^−^^1^ (Scenario 3) and approximately 1.09 × 10^6^ kg CO_2_‐eq batch^−^^1^ (Scenario 4), exceeding even the succinic acid and xylitol pretreatment burdens. We attribute this, tentatively, to the additional chemical loading associated with the HCl neutralization step applied to the NaOH‐rich lignin liquor in this pathway, though this specific mechanism was not directly decomposed in the present analysis and warrants confirmation in future work. The subsequent adhesive activation, methylolation, and synthesis stages carry the highest exergoenvironmental factors observed in this study (0.96–0.998), mirroring the capital‐dominated cost behavior identified for the same units in the exergoeconomic analysis (Section 3.2.4). This convergence indicates that both the cost and environmental burden of adhesive production are governed primarily by reagent and equipment choice rather than operational efficiency, so reducing exergy destruction alone would have limited effect. Since phenol and formaldehyde represent substantial reagent inputs to this pathway, reducing the phenol substitution ratio in favor of a higher lignin fraction, or sourcing bio‐based phenol and formaldehyde alternatives, offer more promising routes to lowering its environmental burden.

### 3.3 Exergoeconomic Analysis

A comparative exergoeconomic analysis was conducted to elucidate how feedstock selection influences cost formation mechanisms across integrated PLA biorefineries co‐producing succinic acid, xylitol, adhesive, and electricity. While both corn stover and sugarcane bagasse systems exhibit similar thermodynamic structures and processing sequences, the magnitude and origin of exergoeconomic penalties differ markedly between the two feedstocks, reflecting variations in exergy destruction levels, capital intensity, and utility requirements. A consolidated comparison of unit exergoeconomic costs of fuel and product is presented in Tables S8‐1, S8‐2, S8‐3, and S8‐4 and illustrated in Table 2. These tables report the complete set of exergoeconomic indicators discussed throughout this section, fuel and product exergy, unit fuel and product cost, exergy destruction, cost of exergy destruction, capital investment cost rate, relative cost difference, and exergoeconomic factor, for every subunit, scenario, and feedstock, and therefore underlie the results presented in Figures 6, 7, 8 (Sections 3.2.2–3.2.4) as well.

**TABLE 2:** Unit exergoeconomic cost of fuel and product for all subunits of all scenarios.

	Unit exergoeconomic cost of fuel (C_f_)		Unit exergoeconomic cost of product (C_p_)	
Process	Corn stover	Bagasse	Corn stover	Bagasse
Alkali pretreatment	6.34	4.38	12.51	9.16
Enzymatic hydrolysis	29.12	25.97	45.78	41.41
Lactic acid fermentation	38.09	35.07	74.87	71.97
Lactic acid purification	76.74	73.90	149.87	144.77
Lactic acid to lactide conversion	148.18	143.15	166.94	161.24
Lactide purification	164.84	159.12	184.11	177.87
PLA polymerization	163.93	158.44	167.38	161.81
PLA purification	154.15	149.06	195.02	188.61
Electricity generation	9.30	6.33	69.46	49.98
SA pretreatment	83.70	78.89	103.08	98.26
SA fermentation	104.28	99.64	202.51	185.80
SA purification	201.75	185.10	344.19	958.85
Xylitol pretreatment	83.70	78.89	102.99	96.13
Xylitol fermentation	106.53	102.53	238.60	239.29
Xylitol purification	240.14	187.70	566.57	288.04
AD‐pretreatment	21.30	15.79	43.80	30.02
AD‐activation	43.91	30.11	46.47	31.76
AD‐methylolation	46.32	31.71	48.80	33.33
AD‐synthesis	22.93	17.18	25.10	18.81

FIGURE 6Investment cost rate and exergy‐destruction cost rate for integrated PLA biorefineries based on corn stover and sugarcane bagasse across (a) Scenario 1, (b) Scenario 2, (c) Scenario 3, and (d) Scenario 4.

**Figure:**
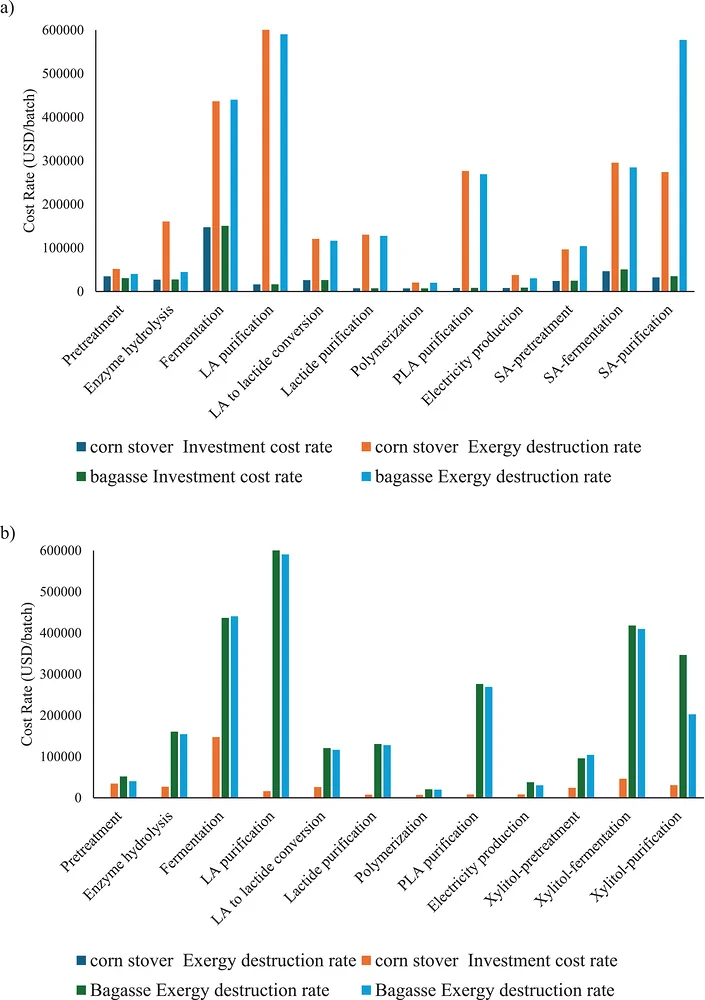

**Figure:**
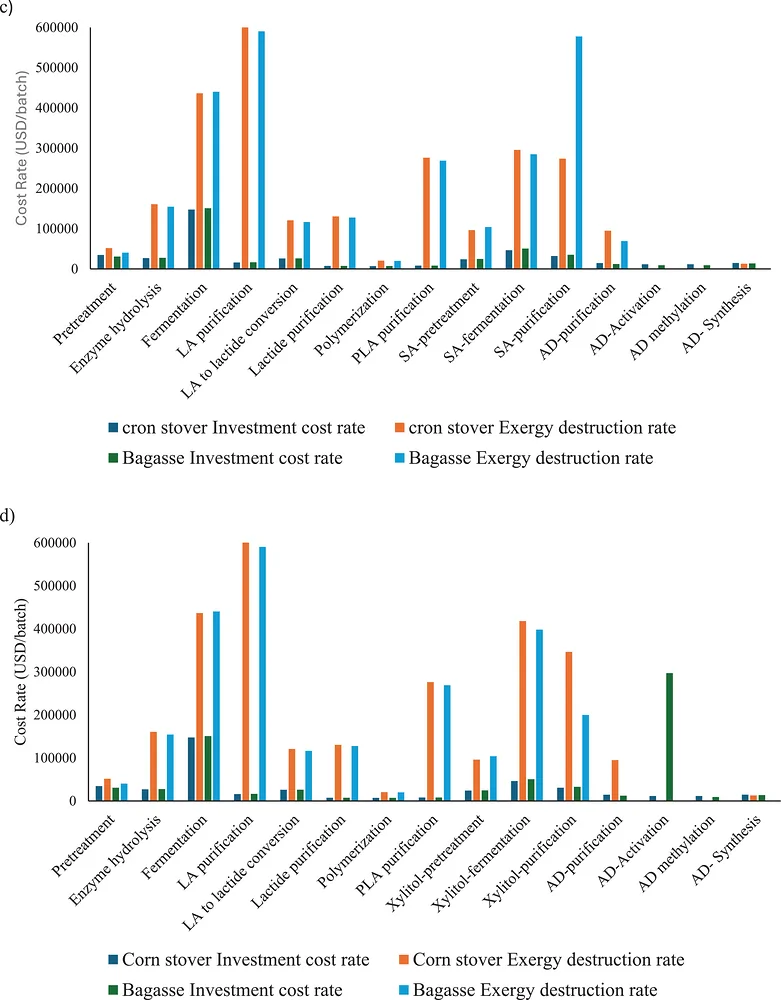

**FIGURE 7:**
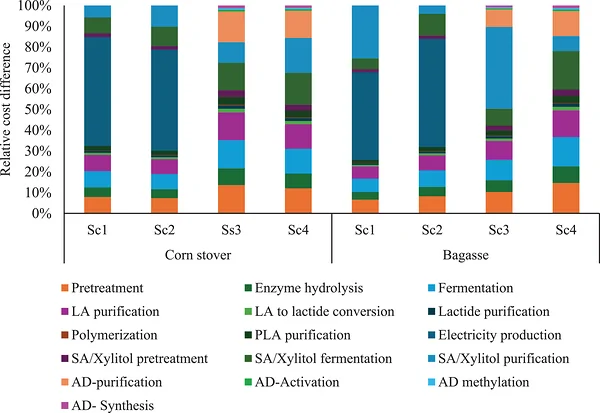
Relative cost difference of all subunits of all scenarios.

**FIGURE 8:**
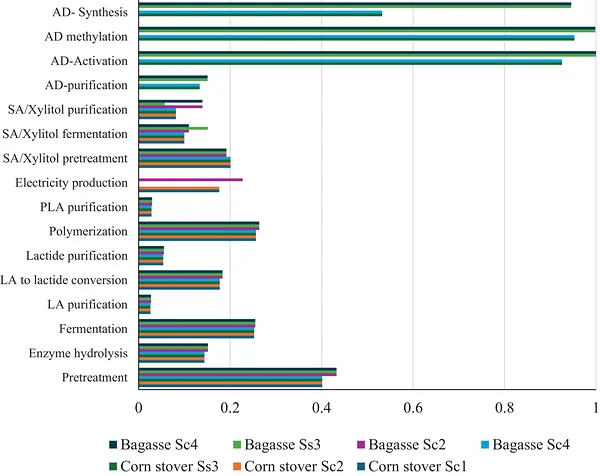
Exergoeconomic factor of all subunits of all scenarios.

#### 3.3.1 Unit Exergoeconomic Cost of Fuel and Product

For both feedstocks and across all scenarios, the baseline PLA production pathway displays a consistent increase in unit exergoeconomic cost toward downstream purification. However, in neither case does the PLA pathway constitute the dominant cost hotspot. Instead, downstream co‐product recovery and upgrading processes exert the strongest influence on product cost formation, confirming that the choice of co‐product strategy governs exergoeconomic performance more strongly than feedstock selection alone.

In Scenario 1 (PLA + succinic acid + electricity), both feedstocks exhibit pronounced cost escalation in succinic acid purification. For corn stover, the unit product cost reaches 344.19 USD GJ^−^^1^, approximately twice that of PLA purification (195.02 USD GJ^−^^1^), driven by high propagation of fuel cost (c_f_  =  201.75 USD GJ^−^^1^). The corresponding input quantities, unit prices, exergy flows, and resulting unit fuel exergy costs (cf) underlying these values, for all major process steps and both feedstocks, are reported in Table S9. In contrast, the bagasse‐based system exhibits a substantially higher unit product cost for the same operation (958.85 USD GJ^−^^1^), despite comparable process configuration. This difference is attributable to higher exergy destruction (3120 GJ batch^−^^1^) and stronger utility‐related cost propagation in the bagasse case, indicating a stronger coupling between thermodynamic irreversibility and economic penalty for this feedstock. This behavior arises from the higher structural carbohydrate content of sugarcane bagasse, which increases fermentable sugar production and intensifies downstream separation requirements. As a result, higher material throughput in the succinic acid purification section increases both exergy destruction and utility‐related cost propagation compared with the corn stover system.

Scenario 2 (PLA + xylitol + electricity) further highlights these differences. In both systems, xylitol purification emerges as the dominant exergoeconomic hotspot; however, the magnitude differs significantly. For corn stover, the unit product cost reaches 566.57 USD GJ^−^^1^, whereas for bagasse it is substantially lower (288.04 USD GJ^−^^1^). In the corn stover system, the high product cost coincides with elevated fuel costs (c_f_  =  240.14 USD GJ^−^^1^), reflecting significant utility and material requirements. In contrast, the bagasse‐based system exhibits moderate exergy destruction (≈ 1079 GJ batch^−^^1^), together with a lower relative cost difference and comparatively moderate fuel cost propagation (c_f_  =  102.53 USD GJ^−^^1^), indicating reduced cost amplification across this unit. Consistent with this behavior, the unit product cost of xylitol pretreatment in the bagasse system remains moderate (cp  =  96.13 USD GJ^−^^1^), reflecting limited cost escalation relative to purification. This divergence demonstrates that similar process configurations can yield markedly different cost magnitudes depending on feedstock‐specific propagation of fuel cost and separation intensity.

Integration of lignin valorization to adhesive (Scenarios 3 and 4) introduces the most pronounced feedstock‐dependent differences. In corn stover–based configurations, adhesive upgrading steps exhibit moderate unit product costs (25–49 USD GJ^−^^1^) alongside elevated fuel costs, indicating cost propagation through high‐value chemical inputs rather than through exergy destruction. AD purification, despite substantial irreversibility, maintains relatively low unit product cost (43.80 USD GJ^−^^1^), demonstrating weak coupling between destruction and economic burden. In contrast, the bagasse‐based adhesive pathway is characterized by consistently low unit product costs for all upgrading steps (30.02, 31.76, 33.33, and 18.81 USD GJ^−^^1^ for purification, activation, methylolation, and synthesis, respectively), with correspondingly low fuel costs. This behavior confirms that cost formation in bagasse‐derived adhesive production is only weakly affected by thermodynamic irreversibility and reflects limited cost amplification across these units. Consequently, adhesive integration does not introduce extreme cost escalation in the bagasse system and remains comparatively stable relative to other co‐product pathways. Scenario 4, combining xylitol and adhesive integration, preserves these trends. In both systems, xylitol purification and, to a lesser extent, adhesive upgrading influence the overall cost structure; however, absolute unit product costs remain significantly higher for the corn stover‐based system, and variability across units is more pronounced. These findings indicate that corn stover–based systems exhibit stronger cost escalation in separation‐intensive operations, whereas bagasse‐based systems show more moderate but still feedstock‐dependent cost behavior.

Overall, both corn stover and sugarcane bagasse biorefineries exhibit similar qualitative exergoeconomic patterns: increasing costs toward downstream purification, dominance of co‐product recovery over PLA synthesis in cost formation, and strong sensitivity to separation‐intensive and capital‐intensive sub‐systems. However, the magnitude and distribution of unit product costs differ between the two feedstocks. Corn stover–based configurations exhibit higher peak costs in separation‐intensive units, whereas bagasse‐based configurations show greater sensitivity to specific process steps such as succinic acid purification. These differences arise from variations in utility demand, capital cost distribution, and propagation of fuel cost, rather than from fundamentally different thermodynamic behavior. From a comparative perspective, both feedstocks remain viable for integrated PLA biorefinery applications, but their exergoeconomic responses to co‐product selection differ significantly, highlighting the importance of process‐specific optimization.

#### 3.3.2 Investment Cost Rate and Exergy Destruction Rate

A comparative evaluation of investment cost rates and exergy‐destruction cost rates was carried out to assess how feedstock selection influences overall exergoeconomic performance in integrated PLA biorefineries co‐producing succinic acid, xylitol, adhesive, and electricity. Although corn stover and sugarcane bagasse systems share similar process structures across all four scenarios, differences are observed in the magnitude, allocation, and driving mechanisms of cost contributions. Across all scenarios, both feedstocks exhibit a consistent pattern in which the cumulative cost of exergy destruction substantially exceeds the total investment cost rate, indicating that thermodynamic irreversibilities dominate overall exergoeconomic burdens irrespective of feedstock. However, bagasse‐based configurations generally show higher total cost levels and greater sensitivity to scenario changes, reflecting higher utility demand, separation intensity, and capital requirements.

In Scenario 1 (PLA + succinic acid + electricity), the corn stover system exhibits a total investment cost rate of 384 887 USD batch^−^^1^, compared with 393 800 USD batch^−^^1^ for bagasse. In both cases, the cumulative cost of exergy destruction is approximately one order of magnitude higher, reaching 2.51 Million USD batch^−^^1^ for corn stover and 2.65 Million USD batch^−^^1^ for bagasse. This behavior arises from the direct proportionality between exergy destruction cost and both *c_f_* and ${\dot{E}}_{D}$, which amplifies cost accumulation in highly irreversible units. As a result, separation‐intensive processes with high exergy destruction and elevated fuel cost propagation cause $c_{f}{\dot{E}}_{D}$ to dominate the overall cost relative to the investment cost rate. While lactic‐acid purification and fermentation dominate destruction‐driven costs for both feedstocks, bagasse shows a more evenly distributed burden across succinic‐acid and lactic‐acid purification, whereas corn stover concentrates a larger fraction of destruction cost in lactic‐acid purification and core fermentation. Investment costs in both systems are dominated by fermentation and succinic‐acid fermentation. In Scenario 2 (PLA + xylitol + electricity), replacing succinic acid with xylitol leaves the overall investment cost rate in the corn stover system essentially unchanged (383 509 USD batch^−^^1^) while increasing cumulative exergy‐destruction costs to 2.70 Million USD batch^−^^1^, driven by xylitol fermentation and purification. In contrast, the bagasse‐based system exhibits only a marginal reduction in total investment cost (391 584 USD batch^−^^1^), while destruction costs remain high (2.51 Million USD batch^−^^1^). Notably, xylitol purification in the bagasse system shows lower destruction cost than succinic‐acid purification but introduces higher capital intensity, indicating a stronger influence of equipment and utility costs on overall cost formation. In Scenario 3, incorporating lignin valorization to adhesive while maintaining succinic acid leads to increased capital requirements for both feedstocks. Corn stover exhibits a moderate rise in investment cost rate (429 209 USD batch^−^^1^), while cumulative exergy‐destruction costs remain comparable to Scenario 1 (2.58 Million USD batch^−^^1^). Adhesive‐related units contribute modestly to total investment and negligibly to destruction‐driven costs, indicating limited exergoeconomic penalty from adhesive integration in the corn stover system. In contrast, the bagasse‐based system shows a more pronounced increase in destruction‐related costs (3.07 million USD batch^−^^1^), suggesting stronger coupling between lignin upgrading and irreversibility. Scenario 4 (PLA + xylitol + adhesive) produces the greatest divergence between feedstocks. For corn stover, the total investment cost rate remains moderate (427 830 USD batch^−^^1^), while cumulative exergy‐destruction costs increase to 2.77 Million USD batch^−^^1^, driven primarily by xylitol fermentation and purification. In contrast, the bagasse‐based configuration exhibits a sharp increase in capital intensity, with the investment cost rate rising to 714 633 USD batch^−^^1^, while cumulative destruction cost decreases slightly to 2.80 Million USD batch^−^^1^. This shift suggests a transition toward capital‐dominated cost formation, particularly in adhesive activation, which contributes negligibly to exergy destruction but significantly to total investment cost.

Overall, both feedstocks exhibit similar qualitative exergoeconomic behavior, with lactic‐acid purification, fermentation, and co‐product recovery dominating destruction‐related costs. However, the relative importance of capital vs. irreversibility differs between the two systems. Corn stover–based configurations exhibit lower variability in investment cost and remain dominated by exergy‐destruction costs across scenarios, whereas bagasse‐based systems show greater sensitivity to co‐product selection, particularly when capital‐intensive upgrading steps such as adhesive integration are introduced. The distribution of investment cost rates and exergy‐destruction cost rates for all process units and scenarios is presented in Figure 6.

#### 3.3.3 Relative Cost Difference

A comparative evaluation of the relative cost difference was carried out for all subunits across Scenarios 1–4 to assess the sensitivity of unit exergoeconomic product costs to improvement in corn stover– and sugarcane bagasse–based biorefineries. This indicator provides insight into the distribution of improvement potential across system components. While both feedstocks exhibit consistent patterns within the baseline PLA production pathway, differences are observed in the magnitude and distribution of improvement potential within co‐product processing sections.

Across all scenarios, the baseline PLA production units show stable and moderate relative cost difference values for both feedstocks. In corn stover configurations, pretreatment (97.41), fermentation (96.56), lactic‐acid (LA) purification (95.29), and enzymatic hydrolysis (57.20) exhibit relatively high sensitivity to improvement, whereas polymerization consistently shows the lowest value (2.11), indicating minimal sensitivity. A similar trend is observed for sugarcane bagasse, where pretreatment (109.32), hydrolysis (59.46), fermentation (105.23), and LA purification (95.91) maintain slightly higher relative cost difference values overall, while polymerization remains largely insensitive (2.13). These results indicate comparable behavior of the core PLA pathway for both feedstocks, with marginally higher cost sensitivity across most upstream units in the bagasse‐based system. Scenario‐specific differences arise primarily from co‐product pathways. In Scenario 1 (PLA + succinic acid + electricity), electricity production shows the highest relative cost difference values for both feedstocks, reaching 646.88 for corn stover and 689.79 for bagasse, identifying it as the primary improvement target. Within the succinic‐acid section, corn stover exhibits comparable contributions from SA fermentation (94.20), and SA purification (70.60), while SA pretreatment remains relatively low (23.16). In contrast, the bagasse‐based system is strongly dominated by SA purification (418.02), followed by SA fermentation (86.48), indicating that separation‐intensive processes exert a much stronger influence on cost sensitivity in the bagasse pathway. In Scenario 2 (PLA + xylitol + electricity), replacing succinic acid with xylitol shifts the dominant improvement potential toward xylitol purification for both feedstocks. For corn stover, xylitol purification exhibits a high relative cost difference value (135.94), exceeding xylitol fermentation (123.97), and pretreatment (≈23.04). In the bagasse‐based system, xylitol fermentation remains the dominant contributor (138.02), followed by xylitol purification (53.45), while electricity production continues to show the highest overall value (689.79). These differences indicate that although the scenario change redistributes the improvement potential, the dominant cost‐sensitivity location differs between feedstocks, with fermentation playing a stronger role in the bagasse pathway and purification remaining more influential in the corn stover system. In Scenarios 3 and 4, which incorporate lignin valorization to adhesive, additional feedstock‐dependent differences emerge. For corn stover, AD purification exhibits relatively high relative cost difference values (105.65), while AD activation (5.83), methylolation (5.34), and synthesis (9.47) remain largely insensitive to improvement. A similar pattern is observed for bagasse, although with slightly lower values for AD purification (90.05) and consistently low values for activation (5.48), methylolation (5.12), and synthesis (9.53), indicating limited sensitivity of these upgrading units. This confirms that adhesive production stages are predominantly insensitive to further cost reduction compared to purification‐intensive units.

Overall, both feedstocks exhibit similar qualitative patterns, with electricity generation and co‐product purification representing the primary contributors to cost sensitivity. However, bagasse‐based systems show substantially higher relative cost difference values in purification‐intensive units, particularly within the succinic‐acid pathway, indicating stronger sensitivity to separation inefficiencies. In contrast, corn stover systems exhibit a more balanced distribution of improvement potential across fermentation and purification stages, reflecting a more distributed cost‐sensitivity profile across the co‐product pathways. Figure 7 compares the relative cost difference values of all subunits in the evaluated corn stover‐ and sugarcane bagasse‐based biorefinery scenarios.

#### 3.3.4 Exergoeconomic Factor

The exergoeconomic factor distinguishes whether the cost burden of each subunit is governed primarily by investment‐related costs (high values) or by exergy‐destruction costs (low values), thereby providing direct guidance for prioritizing either capital‐reduction or energetic‐improvement strategies. Results for corn stover‐ and sugarcane bagasse‐based systems across Scenarios 1–4 are summarized in Figure 8. Across all scenarios, both feedstocks exhibit a consistent exergoeconomic pattern within the baseline PLA production pathway. Pretreatment, fermentation, polymerization, and lactic‐acid‐to‐lactide conversion show moderate‐to‐high exergoeconomic factors for both feedstocks, indicating a dominant contribution of investment‐related costs. In corn stover‐based systems, pretreatment exhibits an exergoeconomic factor of 0.401, while fermentation (0.253), polymerization (0.256), and LA‐to‐lactide conversion (0.177) similarly reflect capital‐dominated cost behavior. A comparable trend is observed for sugarcane bagasse, where pretreatment (0.43), fermentation (0.25), and polymerization (0.26) again show relatively high values. These results indicate that the core conversion and synthesis units behave similarly for both feedstocks and would benefit most from capital‐cost reduction and equipment design optimization rather than from improvements in thermodynamic efficiency.

In contrast, the main purification steps consistently exhibit very low exergoeconomic factors for both feedstocks, highlighting strong dominance of exergy‐destruction‐related costs. Lactic‐acid purification (0.02–0.03), PLA purification (0.02–0.03), and lactide purification (≈ 0.05) display minimal sensitivity to capital reduction and instead require reductions in thermodynamic irreversibilities to achieve meaningful exergoeconomic improvement. Enzymatic hydrolysis exhibits intermediate exergoeconomic factors in both systems (≈ 0.14–0.15), indicating a mixed cost structure in which both energetic and capital‐related improvements may be relevant.

Scenario‐dependent differences arise primarily within the co‐product pathways. In Scenarios 1 and 3 (succinic‐acid route), both feedstocks display low exergoeconomic factors for SA fermentation and SA purification, confirming dominance of destruction‐driven costs. However, the dominance of irreversibility is more pronounced for corn stover, where SA fermentation (0.100) and SA purification (0.0816) exhibit particularly low values. Sugarcane bagasse shows similarly low factors, although with a slightly higher value for SA fermentation ( 0.15), indicating a marginally greater contribution of investment costs in this unit relative to corn stover.

A similar pattern is observed in Scenarios 2 and 4 (xylitol route). For both feedstocks, xylitol fermentation remains strongly destruction‐dominated ( 0.10), while xylitol purification shows slightly higher exergoeconomic factors in the bagasse‐based system (0.13) than in the corn stover system (0.0816), indicating relatively greater sensitivity to capital‐related costs for bagasse in this separation step. Nevertheless, exergy destruction remains the dominant cost driver in xylitol recovery for both feedstocks.

The inclusion of the adhesive (AD) pathway in Scenarios 3 and 4 introduces a pronounced contrast between baseline and upgrading operations. For both feedstocks, AD activation, methylolation, and synthesis exhibit exergoeconomic factors close to unity (≈ 0.93–0.99), indicating overwhelming dominance of investment‐related costs. Consequently, optimization strategies for these units should prioritise equipment sizing, process simplification, and capital cost minimization rather than improvements in energetic efficiency. AD purification represents an exception within the adhesive pathway, displaying moderate exergoeconomic factors (≈ 0.13–0.15) for both feedstocks, suggesting that both capital optimization and reductions in exergy destruction could contribute meaningfully to cost reduction.

Overall, the comparative analysis reveals two clearly defined optimization domains that are largely independent of feedstock choice. Capital‐dominated units such as pretreatment, polymerization, and adhesive upgrading steps should be targeted through investment‐cost reduction and process design improvements, while destruction‐dominated units, particularly purification operations throughout the PLA and co‐product pathways, require focused efforts to reduce thermodynamic irreversibilities. While corn stover and sugarcane bagasse systems exhibit broadly similar qualitative exergoeconomic behavior, corn stover tends to show slightly stronger irreversibility dominance in co‐product purification, whereas bagasse shows marginally higher sensitivity to capital costs in selected separation steps. These findings underscore the importance of tailoring optimization strategies to the dominant cost drivers of each process block rather than applying feedstock‐specific improvements uniformly across the biorefinery.

Downstream purification steps (lactic acid, succinic acid, and xylitol purification) collectively accounted for a substantial share of both exergy destruction and as shown in the exergoeconomic analysis (Table 2). These losses stem from the chemical‐intensive nature of ion‐exchange and solvent‐based separation (e.g., HCl/NaOH regeneration, ethyl acetate use in lactide purification), which introduce exergy losses both through the chemical inputs themselves and through the utility (heating, distillation) required to recover and reuse them. Remedies include membrane‐based separation technologies (e.g., nanofiltration or electrodialysis) to reduce reliance on chemical regenerants, and tighter heat integration (pinch analysis) across the purification train to recover thermal exergy that is currently rejected between sequential unit operations.

## 4 Conclusion

This study presents an integrated exergy, exergoeconomic, and exergoenvironmental assessment of lignocellulosic biorefineries for the co‐production of polylactic acid (PLA) and value‐added co‐products from corn stover and sugarcane bagasse. By systematically evaluating alternative co‐product portfolios, including succinic acid, xylitol, lignin‐based adhesive, and electricity, the study provides a comprehensive understanding of how process configuration and feedstock selection influence thermodynamic performance, cost formation, and overall sustainability. The results demonstrate that co‐product strategy exerts a greater influence on system performance than feedstock choice. Across all configurations, material‐oriented lignin valorization consistently outperforms electricity generation pathways, achieving higher exergy efficiencies, lower exergy destruction, and more effective utilization of biomass resources. In particular, the PLA‐succinic acid‐adhesive configuration emerges as the most favorable option, indicating that directing lignin toward high‐value material applications provides greater sustainability benefits than combustion‐based energy recovery.

The analysis further reveals that the principal thermodynamic and economic limitations of lignocellulosic biorefineries originate from downstream separation and purification processes rather than from the core biochemical conversion pathways. Purification‐intensive operations are responsible for the largest exergy destruction and the strongest propagation of product costs, confirming their role as the dominant system bottlenecks. In contrast, lignin upgrading to adhesive exhibits relatively low thermodynamic penalties but substantial capital‐related cost contributions, highlighting the importance of balancing process efficiency with investment requirements when designing valorization pathways. Although both feedstocks show similar thermodynamic behavior, notable differences arise in their exergoeconomic characteristics. Corn stover‐based systems exhibit more stable cost structures dominated by irreversibility‐related losses, whereas sugarcane bagasse–based systems display greater sensitivity to capital‐intensive process integration. This indicates that feedstock selection influences not only overall performance but also the mechanisms through which costs are generated and distributed. From a process design perspective, three key priorities are identified for improving the sustainability of PLA biorefineries: (i) reducing utility and solvent demand in downstream separation systems, (ii) enhancing heat recovery and process integration to minimize avoidable exergy losses, and (iii) prioritizing lignin valorization routes that maximize material recovery and product value rather than energy generation. Addressing these priorities enables simultaneous thermodynamic, economic, and environmental improvements. Overall, this work demonstrates that the sustainability of lignocellulosic biorefineries is governed primarily by co‐product integration and downstream processing efficiency rather than feedstock selection alone. The integrated assessment framework developed in this study provides a robust basis for designing flexible, resource‐efficient, and economically viable biorefineries aligned with circular bioeconomy objectives. Probabilistic uncertainty and sensitivity analysis of the economic parameters were not included in the present deterministic framework and are identified as a direction for future work.

## Author Contributions

**Reza Hessampour**: writing – original draft, conceptualization, visualization, methodology, formal analysis, data curation, validation, software, resources, project administration, investigation. **Ali Jahanian**: writing – review & editing, conceptualization, validation. **Jerome Ramirez**: writing – review & editing, conceptualization, validation. **Ian O'Hara**: writing – review & editing, validation, supervision, resources, project administration, methodology, investigation, funding acquisition, conceptualization.

## Conflicts of Interest

The author declares no conflicts of interest.

## Supporting information




**Supporting File**: gch270164‐sup‐0001‐SuppMat.docx.

## Data Availability

The data supporting this article have been included as part of the Supplementary Information.
