# Supplementary material for: Exergoeconomic, Exergoenvironmental, and Renewability Assessment of Corn Stover and Sugarcane Bagasse PLA Biorefineries With Co‐Product Valorization
Source: Glob Chall. 2026 Oct 24;10(10):e70164. doi: 10.1002/gch2.70164 (PMC13695154; doi:10.1002/gch2.70164)
Supplement: Supplementary file 1 [file GCH2-10-e70164-s001.docx]

**Exergoeconomic, exergoenvironmental, and renewability assessment of corn stover and sugarcane bagasse PLA biorefineries with co-product valorisation**

**Reza Hessampour ^a,b,d*^, Ali Jahanian ^a, b, c^, Jerome Ramirez ^a, b, c^, Ian O'Hara ^a, b, c, d^**

^a^ School of Mechanical, Medical and Process Engineering, Faculty of Engineering, Queensland University of Technology, Brisbane, Australia

^b^ Centre for Agriculture and Bioeconomy, Faculty of Science, Queensland University of Technology, Brisbane, Australia

^c^ ARC Centre of Excellence in Synthetic Biology, Queensland University of Technology, Brisbane, Australia

^d^ ARC Industrial Transformation Training Centre for Bioplastics and Biocomposites, Queensland University of Technology, Brisbane, Australia

^*^ Corresponding Author:

[reza.hessampour@hdr.qut.edu.au](mailto:reza.hessampour@hdr.qut.edu.au)

r.hesampour@yahoo.com

**SI.1 - Scenario Overview**

Table SI.1 The four scenarios were selected to represent contrasting valorisation strategies for the two principal residual streams generated during PLA production, succinic acid versus xylitol for the xylose-rich stream, and electricity versus lignin-based adhesive for the lignin-rich stream, applied identically to both feedstocks to isolate feedstock effects from co-product strategy effects (see Introduction for full rationale).

**Table SI.1. Overview of evaluated biorefinery scenarios for sugarcane bagasse and corn stover.**

| **Feedstock** | **Scenario** | **Process Technologies** | **Fermentation Bacteria** |
| --- | --- | --- | --- |
| Sugarcane Bagasse | Sc1 | Pretreatment + Electricity + Enzyme hydrolysis + Fermentation + Purification + Polymerization + Succinic acid | *1- Bacillus coagulans 2- Actinobacillus succinogenes* |
|  | Sc2 | Pretreatment + Electricity + Enzyme hydrolysis + Fermentation + Purification + Polymerization + Xylitol | *1- Bacillus coagulans 2- Candida guilliermondii* |
|  | Sc3 | Pretreatment + Adhesive + Enzyme hydrolysis + Fermentation + Purification + Polymerization + Succinic acid | *1- Bacillus coagulans 2- Actinobacillus succinogenes* |
|  | Sc4 | Pretreatment + Adhesive + Enzyme hydrolysis + Fermentation + Purification + Polymerization + Xylitol | *1- Bacillus coagulans 2- Candida guilliermondii* |
| Corn Stover | Sc1 | Pretreatment + Electricity + Enzyme hydrolysis + Fermentation + Purification + Polymerization + Succinic acid | *1- Bacillus coagulans 2- Actinobacillus succinogenes* |
|  | Sc2 | Pretreatment + Electricity + Enzyme hydrolysis + Fermentation + Purification + Polymerization + Xylitol | *1- Bacillus coagulans 2- Candida guilliermondii* |
|  | Sc3 | Pretreatment + Adhesive + Enzyme hydrolysis + Fermentation + Purification + Polymerization + Succinic acid | *1- Bacillus coagulans 2- Actinobacillus succinogenes* |
|  | Sc4 | Pretreatment + Adhesive + Enzyme hydrolysis + Fermentation + Purification + Polymerization + Xylitol | *1- Bacillus coagulans 2- Candida guilliermondii* |

**SI-2-**Process flow diagram of Scenario 1 (succinic acid + electricity co-production), applicable to both corn stover and sugarcane bagasse feedstocks.

**SI.3-** Process flow diagram of Scenario 3 (succinic acid + lignin-based adhesive co-production), applicable to both corn stover and sugarcane bagasse feedstocks.

**SI.3-** Process flow diagram of Scenario 2 (xylitol + electricity co-production), applicable to both corn stover and sugarcane bagasse feedstocks.

**SI.4-** Process flow diagram of Scenario 4 (xylitol + lignin-based adhesive co-production), applicable to both corn stover and sugarcane bagasse feedstocks.

**SI.6-A Key Process Parameters and Economic Assumptions**

Note: The baseline value for each parameter listed below was obtained from one of two sources: (i) process literature specific to the corresponding unit operation, or (ii) standard techno-economic assessment practice for the financial and planning parameters. Feedstock composition, pretreatment, and enzymatic hydrolysis parameters follow Nalawade et al. (2020) (Ref. 45); lactic acid purification parameters follow Bishai et al. (2015) (Ref. 46) and Raya-Tonetti et al. (1999) (Ref. 47); lactide synthesis parameters, dehydration, oligomerisation, thermal depolymerisation, and recrystallisation purification, follow Hu et al. (2017) (Ref. 48), Duarte et al. (2023) (Ref. 49), Sanglard et al. (2012) (Ref. 50), and Tiwari et al. (2024) (Ref. 51); ring-opening polymerisation parameters follow Tiwari et al. (2024) (Ref. 51); succinic acid production and recovery parameters follow Jokodola et al. (2022) (Ref. 52); xylitol fermentation parameters follow Thapa et al. (2022) (Ref. 53), and xylitol purification/crystallisation parameters follow Wei et al. (2010) (Ref. 54); lignin-to-adhesive conversion parameters follow Ai et al. (2021) (Ref. 55). Financial and planning parameters (discount rate, plant lifetime, maintenance factor) follow standard techno-economic assessment practice, consistent with the exergoeconomic framework of Amid et al. (2021) (Ref. 25). Parameters not directly attributable to a specific cited source (e.g., plant operating schedule, functional-unit basis, and solvent/catalyst recovery efficiencies) reflect engineering assumptions made by the authors for this study. Full bibliographic details for each source are provided in the reference list of the main manuscript.

**Table SI.6-A. Baseline process parameters and economic assumptions applied across all scenarios.**

| **Parameter** | **Value / Baseline** |
| --- | --- |
| Bagasse composition: Ash (insoluble solids) | 2 % |
| Cellulose | 43 % |
| Hemicellulose | 31 % |
| Lignin | 23 % |
| Extractives | 1 % |
| Corn Stover composition: Insoluble solids (ash) | 5.5 % |
| Cellulose | 35.0 % |
| Hemicellulose | 22.0 % |
| Lignin | 16.0 % |
| Extractives | 21.5 % |
| Pretreatment temperature | 100 °C |
| Solid loading | 20 % |
| NaOH concentration | 0.5 M |
| Residence time | 120 min |
| Filtration flux | 200 L/m²·h |
| Enzyme loading | 20 FPU/g |
| Hydrolysis solids loading | 20 % |
| Hydrolysis time | 72 h |
| Glucose conversion rate | 86 % |
| Xylose conversion rate | 86 % |
| Fermentation time | 30 h |
| Glucose conversion to LA | 92 % |
| Fermentation temperature | 50 °C |
| Depolymerization temperature | 220 °C |
| LA→Lactide conversion yield | 90 % |
| Depolymerization time | 3 h |
| Lactide purification temperature | 80 °C |
| Lactide purity | 99 % |
| Ethyl acetate recycling rate | 99 % |
| Ethyl acetate ratio (w/v) | 1:1.5 |
| Polymerization: Toluene (w/v) | 1.5 |
| Monomer conversion | 99 % |
| Stirring time | 12 h |
| SA fermentation time | 30 h |
| SA conversion rate | 50 % |
| Vacuum distillation temperature | 60 °C |
| Crystallisation temperature | 4 °C |
| SA drying temperature/time | 70 °C / 4 h |
| Xylitol conversion rate | 43 % |
| Xylitol fermentation time | 72 h |
| Xylitol fermentation temperature | 30 °C |
| Formaldehyde solution | 0.27 (% w/v) |
| Adhesive synthesis: phenol+HCHO→resin conversion | 90 % |
| ML reaction conversion | 80 % |
| Electricity price | 0.19095 USD/kWh |
| Heat price | 0.03819 USD/kWh |
| Enzyme price | 3.00 USD/kg |
| Zn-(Phe)₂ price | 5.00 USD/kg |
| Inflation rate | 3 % |
| Discount rate | 10 % |
| Plant lifetime | 30 years |

**SI.6-B - Material Quantities per 1000 kg PLA**
All material quantities are normalised to a functional unit (FU) of 1000 kg PLA, consistent with the exergy analysis basis defined in the main text.

**Table SI.6-B. Key material quantities per functional unit for bagasse and corn stover.**

| **Materials / Nutrients** | **Bagasse (kg/FU)** | **Corn Stover (kg/FU)** |
| --- | --- | --- |
| **LA Fermentation** |  |  |
| Corn Steep Liquor | 430.06 | 417.70 |
| Ammonium Sulfate | 13.64 | 13.24 |
| CaCO₃ | 629.36 | 611.27 |
| **LA Inoculum Preparation** |  |  |
| Glucose | 52.45 | 50.94 |
| Yeast extract | 10.49 | 10.19 |
| **SA Fermentation** |  |  |
| Yeast extract | 16.73 | 14.59 |
| Na₂HPO₄ | 1.00 | 0.88 |
| NaH₂PO₄ | 4.68 | 4.08 |
| NaCl | 3.35 | 2.92 |
| K₂HPO₄ | 5.02 | 4.38 |
| MgCl₂·2H₂O | 0.67 | 0.58 |
| CaCl₂·2H₂O | 0.67 | 0.58 |
| MgCO₃ | 66.92 | 58.35 |
| **SA Inoculum Preparation** |  |  |
| Pancreatic digest of casein | 5.69 | 4.96 |
| Soy peptone | 1.00 | 0.88 |
| Glucose | 0.84 | 0.73 |
| NaCl | 1.67 | 1.46 |
| KH₂PO₄ | 0.84 | 0.73 |
| **Xylitol Fermentation** |  |  |
| Rice Bran Extract | 133.83 | 116.69 |
| Ammonium Sulfate | 6.69 | 5.83 |
| Calcium Chloride | 0.33 | 0.29 |
| **Xylitol Inoculum Preparation** |  |  |
| Rice Bran Extract | 13.38 | 11.66 |
| Ammonium Sulfate | 0.66 | 0.58 |
| Calcium Chloride | 0.03 | 0.02 |
| Xylose | 10.03 | 8.75 |

**SI.6-C -Stream Exergy Model Inputs at Major Process Boundaries**

**Table SI.6-C. Key component masses and exergy model inputs at major process boundaries for sugarcane bagasse and corn stover.**

| **Process Boundary** | **T (°C)** | **P (bar)** | **Key Components - Bagasse (kg/batch, selected)** | **Key Components — Corn Stover (kg/batch, selected)** | **Exergy Model (Eq. reference)** |
| --- | --- | --- | --- | --- | --- |
| 1 — Feedstock Input (dry basis) | 25 | 1.01 | Cellulose: 496,936 \| Hemi: 358,256 \| Lignin: 265,803 \| Ash: 23,113 \| Total dry: 1,144,108 kg | Cellulose: 493,489 \| Hemi: 310,193 \| Lignin: 225,595 \| Ash: 77,548 \| Total dry: 1,409,967 kg | Eq. 5–6 (Song et al.) for each component; Eq. 4 for mixture. Ash treated as inert (ε = 0). |
| 2 — Pretreatment (0.5 M NaOH, 100 °C, 120 min, 20% w/v) | 100 | 1.01 | NaOH: 78,264 \| Water: 3,834,944 \| Electricity: 14,841 kWh \| Heat: 378,053 kWh → Total input: 24,086 GJ \| Output solid: 14,866 GJ \| φ = 98.3% | NaOH: 95,486 \| Water: 4,678,821 \| Electricity: 18,104 kWh \| Heat: 461,244 kWh → Total input: 22,166 GJ \| Output solid: 14,001 GJ \| φ = 97.7% | Szargut: ε_NaOH=74.9 kJ/mol, ε_H₂O=0.9 kJ/mol. Physical exergy Eq. 1–3. Electricity Eq. 7 (Carnot). Heat Eq. 6. |
| 3 — Enzyme Hydrolysis (Cellic CTec2, 50 °C, 72 h, 20% w/v) | 50 | 1.01 | Water: 3,092,419 \| Enzyme: 104,474 \| Elec: 2,764 kWh \| Heat: 98,699 kWh → Total: 17,738 GJ \| Output: 11,789 GJ \| φ = 83.5% | Water: 2,988,313 \| Enzyme: 100,957 \| Elec: 2,663 kWh \| Heat: 95,376 kWh → Total: 16,776 GJ \| Output: 11,262 GJ \| φ = 83.5% | Enzyme Eq. 5–6 (protein proxy). Water Szargut. IP = 1.33 GJ/FU for both feedstocks. |
| 4 — LA Fermentation (B. coagulans, 50 °C, pH 7.0, 30 h) | 50 | 1.01 | Glucose: 495,864 \| CLS: 150,125 \| CaCO₃: 219,695 \| FeSO₄: 4,760 → LA output: 456,195 kg \| Total in: 28,571 GJ \| Out: 16,016 GJ \| φ = 95.4% | Glucose: 492,424 \| CLS: 144,800 \| CaCO₃: 211,903 \| FeSO₄: 4,591 → LA output: 453,030 kg \| Total in: 27,338 GJ \| Out: 15,879 GJ \| φ = 98.8% | LA Eq. 5–6 (C=39.99%, H=6.71%, O=53.30%). CaCO₃ Szargut ε=829.5 kJ/mol. Xylose exits unconsumed to SA/xylitol pathway. |
| 5 — LA Purification (centrifugation → UF → IEX, HCl elution) | 25 | 1.01 | HCl: 209,166 kg \| Water: 40,541 kg \| Elec: 1,328 kWh → Total in: 16,553 GJ \| Out: 8,563 GJ \| φ = 92.7% \| c_f = 73.90 USD/GJ | HCl: 207,715 kg \| Water: 39,103 kg \| Elec: 4,495 kWh → Total in: 16,411 GJ \| Out: 8,511 GJ \| φ = 89.8% \| c_f = 76.74 USD/GJ | HCl binary mixture Eq. 4 with Szargut ε_HCl. Dominant irreversibility source in both systems. |
| 6 — LA→Lactide (dehydration 80°C → oligomer 150°C → depoly 220°C, ZnO) | 220 | ~0.03 | LA: 456,195 \| ZnO: 38.8 \| Heat: 110,462 kWh total → In: 8,690 GJ \| Out: 7,877 GJ \| φ = 90.6% \| IP = 0.22 GJ/FU | LA: 453,030 \| ZnO: 38.6 \| Heat: 109,697 kWh total → In: 8,637 GJ \| Out: 7,822 GJ \| φ = 90.6% \| IP = 0.22 GJ/FU | Lactide Eq. 5–6 (C₆H₈O₄: C=50%, H=5.56%, O=44.44%). Three sub-steps modelled individually. |
| 7 — Lactide Purification (recrystallisation, EtOAc, 80°C) | 80 | 1.01 | Crude lactide \| EtOAc: 4,741 kg \| Heat: 10,302 kWh → In: 8,008 GJ \| Out: 7,206 GJ \| φ = 90.0% \| c_f = 159.12 USD/GJ | Crude lactide \| EtOAc: 4,708 kg \| Heat: 10,231 kWh → In: 7,947 GJ \| Out: 7,156 GJ \| φ = 90.0% \| c_f = 164.84 USD/GJ | EtOAc Eq. 5–6. Recycled at 99%; modelled as negligible net exergy loss. |
| 8 — Ring-Opening Polymerisation (Zn-(Phe)₂, toluene, 12 h) | 150 | ~1 | L-Lactide \| Toluene: 22,425 kg \| Zn-(Phe)₂: 95.5 kg → In: 8,187 GJ \| Out: 8,061 GJ \| φ = 98.5% \| c_f = 158.44 USD/GJ | L-Lactide \| Toluene: 22,269 kg \| Zn-(Phe)₂: 94.8 kg → In: 8,131 GJ \| Out: 8,005 GJ \| φ = 98.5% \| c_f = 163.93 USD/GJ | Toluene Eq. 5–6 (C₇H₈). Recovered by closed-loop distillation; modelled as full recovery. |
| 9 — PLA Purification (DCM precipitation, 5% HCl/MeOH, drying) | 25 | 1.01 | HCl: 799 kg \| MeOH: 32,616 kg \| Water: 68,205 kg → In: 8,814 GJ \| Out: 7,009 GJ \| φ = 90.7% \| c_f = 149.06 USD/GJ | HCl: 794 kg \| MeOH: 32,390 kg \| Water: 67,455 kg → In: 8,753 GJ \| Out: 6,960 GJ \| φ = 90.7% \| c_f = 154.15 USD/GJ | MeOH Eq. 5–6 (C=37.49%, H=12.58%, O=49.93%). PLA treated as lactic acid repeat unit for chemical exergy. |
| 10 — SA Co-production (A. succinogenes, 37 °C, 30 h) [Sc1, Sc3] | 37 | 1.01 | Xylose: 210,942 kg \| Yeast ext: 16,730 kg \| MgCO₃: 66,920 kg → SA in: 7,959 GJ \| SA ferm in: 6,752 GJ \| SA out: 3,912 GJ \| φ_ferm = 57.7% | Xylose: 182,642 kg \| Yeast ext: 16,340 kg \| MgCO₃: 65,350 kg → SA in: 7,376 GJ \| SA ferm in: 6,319 GJ \| SA out: 3,507 GJ \| φ_ferm = 55.1% | SA Eq. 5–6 (C=40.67%, H=5.12%, O=54.21%). Highest exergy destruction rate in Sc1/Sc3 after electricity generation. |
| 11 — Xylitol Co-production (Candida sp., 30 °C, 72 h) [Sc2, Sc4] | 30 | 1.01 | Xylose: 210,942 kg \| Rice bran ext: 133,830 kg → Xylitol in: 7,959 GJ \| Xylitol ferm in: 7,363 GJ \| Out: 3,414 GJ \| φ_ferm = 47.5% | Xylose: 182,642 kg \| Rice bran ext: 130,690 kg → Xylitol in: 7,376 GJ \| Xylitol ferm in: 6,854 GJ \| Out: 3,295 GJ \| φ_ferm = 44.0% | Xylitol Eq. 5–6 (C=39.47%, H=7.94%, O=52.59%). Lowest efficiencies in both systems due to dilute-sugar irreversibilities. |
| 12 — Electricity Generation (lignin combustion) [Sc1, Sc2] | ~800 | 1.01 | Lignin: 204,934 kg \| HV = 17.5 MJ/kg \| η = 25% → E_ch = 5,674 GJ \| Electricity out: 896,586 MJ \| φ = 15.8% \| Ex_D = 4,777 GJ | Lignin: 173,934 kg \| HV = 17.5 MJ/kg \| η = 25% → E_ch = 4,816 GJ \| Electricity out: 760,959 MJ \| φ = 15.8% \| Ex_D = 4,055 GJ | Lignin Eq. 5–6 (C=65.07%, H=6.07%, O=28.86%). Largest single source of irreversibility in Sc1/Sc2 for both feedstocks. |
| 13 — Lignin Adhesive (purif. → activation 80°C → methylolation 60°C → synthesis) [Sc3, Sc4] | 60–80 | 1.01 | Lignin: 204,934 kg \| Formaldehyde: 129,479 kg \| Phenol: 225,427 kg \| NaOH: 36,888 kg → AD purif in: 10,110 GJ \| AD synth out: 15,012 GJ \| φ_synth = 95.9% | Lignin: 173,934 kg \| Formaldehyde: 110,250 kg \| Phenol: 191,500 kg \| NaOH: 31,355 kg → AD purif in: 9,319 GJ \| AD synth out: 12,741 GJ \| φ_synth = 95.9% | AD activation, methylation, synthesis: φ > 99%; capital-dominated (f_b > 0.95). AD purification: φ = 84–87%; destruction-dominated. |

**SI.7 - Capital Expenditure (CAPEX) by Unit Operation**

Table SI.7 reports the purchased equipment costs and total installed CAPEX for each process unit, for both sugarcane bagasse and corn stover. Installation (20%), instrumentation (26%), piping (31%), electrical (15%), buildings (29%), yard improvements (10%), service facilities (29%), construction (34%), contingencies (37%), and contractor fees (19%) are applied to purchased equipment cost per Kobe (1958) and Towler & Sinnott (2021).

**Table SI.7. Sector-wise CAPEX breakdown by process unit for sugarcane bagasse and corn stover (selected columns; USD per batch).**

| **Process Unit** | **Bagasse Purchase Cost (USD)** | **Corn Stover Purchase Cost (USD)** | **Installation 20%** | **Piping 31%** | **Buildings 29%** | **Constr. 34%** | **Conting. 37%** | **Total CAPEX** |
| --- | --- | --- | --- | --- | --- | --- | --- | --- |
| Pretreatment | 4,676,819 | 4,500,000 | 935,364 | 1,449,814 | 1,356,278 | 1,590,118 | 1,730,423 | **16,368,867** |
| Enzyme hydrolysis | 4,185,834 | 4,030,000 | 837,167 | 1,297,609 | 1,213,892 | 1,423,184 | 1,548,759 | **14,650,420** |
| Inoculum & fermentation | 22,904,349 | 22,050,000 | 4,580,870 | 7,100,348 | 6,642,261 | 7,787,479 | 8,474,609 | **80,165,222** |
| LA & xylose separation | 2,503,701 | 2,410,000 | 500,740 | 776,147 | 726,073 | 851,258 | 926,369 | **8,762,954** |
| LA→Lactide conversion | 3,971,381 | 3,820,000 | 794,276 | 1,231,128 | 1,151,701 | 1,350,270 | 1,469,411 | **13,899,834** |
| Lactide purification | 1,131,903 | 1,090,000 | 226,381 | 350,890 | 328,252 | 384,847 | 418,804 | **3,961,662** |
| Polymerization | 1,087,906 | 1,047,000 | 217,581 | 337,251 | 315,493 | 369,888 | 402,525 | **3,807,669** |
| PLA purification | 1,240,422 | 1,194,000 | 248,084 | 384,531 | 359,722 | 421,744 | 458,956 | **4,341,478** |
| Lignin → electricity | 1,352,767 | 1,302,000 | 270,553 | 419,358 | 392,303 | 459,941 | 500,524 | **4,734,686** |
| SA: Batch distillation | 3,753,404 | 3,613,000 | 750,681 | 1,163,555 | 1,088,487 | 1,276,157 | 1,388,759 | **13,136,914** |
| SA: Inoculum & ferm. | 7,689,289 | 7,403,000 | 1,537,858 | 2,383,680 | 2,229,894 | 2,614,358 | 2,845,037 | **26,912,513** |
| SA: Purification | 5,327,402 | 5,129,000 | 1,065,480 | 1,651,495 | 1,544,947 | 1,811,317 | 1,971,139 | **18,645,908** |
| Xylitol: Batch distill. | 3,753,404 | 3,613,000 | 750,681 | 1,163,555 | 1,088,487 | 1,276,157 | 1,388,759 | **13,136,914** |
| Xylitol: Inoculum & ferm. | 7,689,289 | 7,403,000 | 1,537,858 | 2,383,680 | 2,229,894 | 2,614,358 | 2,845,037 | **26,912,513** |
| Xylitol: Purification | 4,990,787 | 4,805,000 | 998,157 | 1,547,144 | 1,447,328 | 1,696,868 | 1,846,591 | **17,467,754** |
| AD purification | 1,865,683 | 1,796,000 | 373,137 | 578,362 | 541,048 | 634,332 | 690,303 | **6,529,891** |
| AD activation | 1,374,997 | 1,323,000 | 274,999 | 426,249 | 398,749 | 467,499 | 508,749 | **4,812,488** |
| AD methylation | 1,382,334 | 1,330,000 | 276,467 | 428,523 | 400,877 | 469,993 | 511,463 | **4,838,168** |
| AD synthesis | 2,056,984 | 1,980,000 | 411,397 | 637,665 | 596,525 | 699,375 | 761,084 | **7,199,445** |

**SI.8- Exergoeconomic Analysis Summary**

Tables SI.8-1 to SI.8-4 present the complete exergoeconomic balance for all four scenarios and both feedstocks. Columns: Ex_in = fuel exergy (GJ/FU); Ex_out = product exergy (GJ/FU); rc = relative cost difference; Ex_D = exergy destruction (GJ/FU); C_D = cost rate of exergy destruction (USD/batch); Ż = capital cost rate (USD/batch); f_c = exergoeconomic factor; IP = improvement potential (GJ/FU). Economic parameters: interest rate 10%, plant lifetime 30 years, operating hours 7,920 h/yr.

**Table SI.8-1. Exergoeconomic summary - Scenario 1 (PLA + Electricity + Succinic Acid).**

| **Subunit** | **Ex_in (GJ/FU)** | **Ex_out (GJ/FU)** | **rc** | **Ex_D (GJ/FU)** | **C_D (USD/batch)** | **Ż (USD/batch)** | **f_c** | **IP (GJ/FU)** |
| --- | --- | --- | --- | --- | --- | --- | --- | --- |
| **— Sugarcane Bagasse —** |  |  |  |  |  |  |  |  |
| Pretreatment | 24085.77 | 14866.01 | 109.32 | 9219.77 | 40367 | 30785 | 0.43 | 0.034 |
| Enzyme hydrolysis | 17737.81 | 11789.29 | 59.46 | 5948.52 | 154462 | 27553 | 0.15 | 1.335 |
| Fermentation | 28571.03 | 16016.30 | 105.23 | 12554.73 | 440294 | 150768 | 0.25 | 0.708 |
| LA purification | 16552.62 | 8563.11 | 95.91 | 7989.51 | 590389 | 16481 | 0.03 | 0.498 |
| LA→Lactide conversion | 8689.82 | 7876.74 | 12.64 | 813.07 | 116406 | 26142 | 0.18 | 0.223 |
| Lactide purification | 8008.40 | 7205.70 | 11.79 | 802.70 | 127721 | 7451 | 0.05 | 0.229 |
| Polymerization | 8187.37 | 8061.22 | 2.13 | 126.15 | 19987 | 7161 | 0.26 | 0.006 |
| PLA purification | 8813.94 | 7008.93 | 26.53 | 1805.00 | 269045 | 8165 | 0.03 | 0.222 |
| Electricity generation | 4815.74 | 760.96 | 689.79 | 4777.47 | 30234 | 8905 | 0.22 | 9.918 |
| SA pretreatment | 7958.81 | 6641.02 | 24.56 | 1317.80 | 103961 | 24707 | 0.19 | 0.523 |
| SA fermentation | 6751.53 | 3893.00 | 86.48 | 2858.53 | 284812 | 50615 | 0.15 | 3.697 |
| SA purification | 3912.13 | 791.79 | 418.01 | 3120.34 | 577583 | 35068 | 0.05 | 1.527 |
| **— Corn Stover —** |  |  |  |  |  |  |  |  |
| Pretreatment | 22165.71 | 14001.17 | 97.41 | 8164.53 | 51740 | 34687 | 0.40 | 0.034 |
| Enzyme hydrolysis | 16776.27 | 11261.65 | 57.20 | 5514.62 | 160604 | 26993 | 0.14 | 1.335 |
| Fermentation | 27338.27 | 15878.64 | 96.56 | 11459.62 | 436518 | 147537 | 0.25 | 0.708 |
| LA purification | 16411.05 | 8511.01 | 95.29 | 7900.04 | 606270 | 16128 | 0.03 | 0.498 |
| LA→Lactide conversion | 8636.84 | 7821.99 | 12.66 | 814.85 | 120743 | 26033 | 0.18 | 0.223 |
| Lactide purification | 7947.12 | 7155.72 | 11.69 | 791.40 | 130454 | 7420 | 0.05 | 0.229 |
| Polymerization | 8130.58 | 8005.30 | 2.11 | 125.28 | 20536 | 7131 | 0.26 | 0.006 |
| PLA purification | 8752.79 | 6960.32 | 26.51 | 1792.48 | 276316 | 8131 | 0.03 | 0.222 |
| Electricity generation | 4815.74 | 760.96 | 646.88 | 4054.78 | 37709 | 8070 | 0.18 | 9.918 |
| SA pretreatment | 7375.96 | 6223.29 | 23.16 | 1152.67 | 96479 | 24151 | 0.20 | 0.523 |
| SA fermentation | 6318.85 | 3483.02 | 94.20 | 2835.83 | 295721 | 46424 | 0.14 | 3.697 |
| SA purification | 3507.34 | 2149.38 | 70.60 | 1357.96 | 273975 | 32184 | 0.10 | 1.527 |

**Table SI.8-2. Exergoeconomic summary - Scenario 2 (PLA + Electricity + Xylitol).**

| **Subunit** | **Ex_in** | **Ex_out** | **rc** | **Ex_D** | **C_D** | **Ż** | **f_c** | **IP** |
| --- | --- | --- | --- | --- | --- | --- | --- | --- |
| **— Sugarcane Bagasse —** |  |  |  |  |  |  |  |  |
| Pretreatment | 24085.77 | 14866.01 | 109.32 | 9219.77 | 40367 | 30785 | 0.43 | 0.034 |
| Enzyme hydrolysis | 17737.81 | 11789.29 | 59.46 | 5948.52 | 154462 | 27553 | 0.15 | 1.335 |
| Fermentation | 28571.03 | 16016.30 | 105.23 | 12554.73 | 440294 | 150768 | 0.25 | 0.708 |
| LA purification | 16552.62 | 8563.11 | 95.91 | 7989.51 | 590389 | 16481 | 0.03 | 0.498 |
| LA→Lactide conversion | 8689.82 | 7876.74 | 12.64 | 813.07 | 116406 | 26142 | 0.18 | 0.223 |
| Lactide purification | 8008.40 | 7205.70 | 11.79 | 802.70 | 127721 | 7451 | 0.05 | 0.229 |
| Polymerization | 8187.37 | 8061.22 | 2.13 | 126.15 | 19987 | 7161 | 0.26 | 0.006 |
| PLA purification | 8813.94 | 7008.93 | 26.53 | 1805.00 | 269045 | 8165 | 0.03 | 0.222 |
| Electricity generation | 4815.74 | 760.96 | 689.79 | 4777.47 | 30234 | 8905 | 0.22 | 9.918 |
| Xylitol pretreatment | 7958.81 | 6641.21 | 21.86 | 1317.60 | 103945 | 24707 | 0.19 | 0.523 |
| Xylitol fermentation | 7362.82 | 3366.53 | 138.02 | 3996.30 | 398173 | 50615 | 0.11 | 5.490 |
| Xylitol purification | 3413.87 | 2334.33 | 53.45 | 1079.54 | 199825 | 32852 | 0.13 | 2.998 |
| **— Corn Stover —** |  |  |  |  |  |  |  |  |
| Pretreatment | 22165.71 | 14001.17 | 97.41 | 8164.53 | 51740 | 34687 | 0.40 | 0.034 |
| Enzyme hydrolysis | 16776.27 | 11261.65 | 57.20 | 5514.62 | 160604 | 26993 | 0.14 | 1.335 |
| Fermentation | 27338.27 | 15878.64 | 96.56 | 11459.62 | 436518 | 147537 | 0.25 | 0.708 |
| LA purification | 16411.05 | 8511.01 | 95.29 | 7900.04 | 606270 | 16128 | 0.03 | 0.498 |
| LA→Lactide conversion | 8636.84 | 7821.99 | 12.66 | 814.85 | 120743 | 26033 | 0.18 | 0.223 |
| Lactide purification | 7947.12 | 7155.72 | 11.69 | 791.40 | 130454 | 7420 | 0.05 | 0.229 |
| Polymerization | 8130.58 | 8005.30 | 2.11 | 125.28 | 20536 | 7131 | 0.26 | 0.006 |
| PLA purification | 8752.79 | 6960.32 | 26.51 | 1792.48 | 276316 | 8131 | 0.03 | 0.222 |
| Electricity generation | 4815.74 | 760.96 | 646.88 | 4054.78 | 37709 | 8070 | 0.18 | 9.918 |
| Xylitol pretreatment | 7375.96 | 6229.11 | 23.04 | 1146.85 | 95992 | 24151 | 0.20 | 1.092 |
| Xylitol fermentation | 6853.58 | 3254.65 | 123.97 | 3598.93 | 383401 | 46424 | 0.11 | 5.490 |
| Xylitol purification | 3295.17 | 1451.00 | 135.94 | 1844.17 | 442852 | 30805 | 0.06 | 2.998 |

**Table SI.8-3. Exergoeconomic summary - Scenario 3 (PLA + Adhesive + Succinic Acid).**

| **Subunit** | **Ex_in** | **Ex_out** | **rc** | **Ex_D** | **C_D** | **Ż** | **f_c** | **IP** |
| --- | --- | --- | --- | --- | --- | --- | --- | --- |
| **— Sugarcane Bagasse —** |  |  |  |  |  |  |  |  |
| Pretreatment | 24085.77 | 14866.01 | 109.32 | 9219.77 | 40367 | 30785 | 0.43 | 0.034 |
| Enzyme hydrolysis | 17737.81 | 11789.29 | 59.46 | 5948.52 | 154462 | 27553 | 0.15 | 1.335 |
| Fermentation | 28571.03 | 16016.30 | 105.23 | 12554.73 | 440294 | 150768 | 0.25 | 0.708 |
| LA purification | 16552.62 | 8563.11 | 95.91 | 7989.51 | 590389 | 16481 | 0.03 | 0.498 |
| LA→Lactide conversion | 8689.82 | 7876.74 | 12.64 | 813.07 | 116406 | 26142 | 0.18 | 0.223 |
| Lactide purification | 8008.40 | 7205.70 | 11.79 | 802.70 | 127721 | 7451 | 0.05 | 0.229 |
| Polymerization | 8187.37 | 8061.22 | 2.13 | 126.15 | 19987 | 7161 | 0.26 | 0.006 |
| PLA purification | 8813.94 | 7008.93 | 26.53 | 1805.00 | 269045 | 8165 | 0.03 | 0.222 |
| SA pretreatment | 7958.81 | 6641.02 | 24.56 | 1317.80 | 103961 | 24707 | 0.19 | 0.523 |
| SA fermentation | 6751.53 | 3893.00 | 86.48 | 2858.53 | 284812 | 50615 | 0.15 | 3.697 |
| SA purification | 3912.13 | 791.79 | 418.01 | 3120.34 | 577583 | 35068 | 0.05 | 1.527 |
| AD purification | 10110.15 | 5728.99 | 90.05 | 4381.16 | 69196 | 12281 | 0.15 | 1.238 |
| AD activation | 5742.49 | 5729.00 | 5.48 | 13.49 | 406 | 9051 | 0.95 | 0.000 |
| AD methylation | 5784.06 | 5774.83 | 5.12 | 9.23 | 293 | 9099 | 0.97 | 0.000 |
| AD synthesis | 15654.76 | 15011.55 | 9.53 | 643.21 | 11047 | 13540 | 0.55 | 0.069 |
| **— Corn Stover —** |  |  |  |  |  |  |  |  |
| Pretreatment | 22165.71 | 14001.17 | 97.41 | 8164.53 | 51740 | 34687 | 0.40 | 0.034 |
| Enzyme hydrolysis | 16776.27 | 11261.65 | 57.20 | 5514.62 | 160604 | 26993 | 0.14 | 1.335 |
| Fermentation | 27338.27 | 15878.64 | 96.56 | 11459.62 | 436518 | 147537 | 0.25 | 0.708 |
| LA purification | 16411.05 | 8511.01 | 95.29 | 7900.04 | 606270 | 16128 | 0.03 | 0.498 |
| LA→Lactide conversion | 8636.84 | 7821.99 | 12.66 | 814.85 | 120743 | 26033 | 0.18 | 0.223 |
| Lactide purification | 7947.12 | 7155.72 | 11.69 | 791.40 | 130454 | 7420 | 0.05 | 0.229 |
| Polymerization | 8130.58 | 8005.30 | 2.11 | 125.28 | 20536 | 7131 | 0.26 | 0.006 |
| PLA purification | 8752.79 | 6960.32 | 26.51 | 1792.48 | 276316 | 8131 | 0.03 | 0.222 |
| SA pretreatment | 7375.96 | 6223.29 | 23.16 | 1152.67 | 96479 | 24151 | 0.20 | 0.523 |
| SA fermentation | 6318.85 | 3483.02 | 94.20 | 2835.83 | 295721 | 46424 | 0.14 | 3.697 |
| SA purification | 3507.34 | 2149.38 | 70.60 | 1357.96 | 273975 | 32184 | 0.10 | 1.527 |
| AD purification | 9318.65 | 4864.39 | 105.65 | 4454.26 | 94876 | 14594 | 0.13 | 1.238 |
| AD activation | 4885.49 | 4864.39 | 5.83 | 21.10 | 927 | 11524 | 0.93 | 0.000 |
| AD methylation | 4915.67 | 4903.29 | 5.34 | 12.38 | 574 | 11548 | 0.95 | 0.000 |
| AD synthesis | 13304.51 | 12740.76 | 9.47 | 563.75 | 12925 | 14726 | 0.53 | 0.069 |

**Table SI.8-4. Exergoeconomic summary - Scenario 4 (PLA + Adhesive + Xylitol).**

| **Subunit** | **Ex_in** | **Ex_out** | **rc** | **Ex_D** | **C_D** | **Ż** | **f_c** | **IP** |
| --- | --- | --- | --- | --- | --- | --- | --- | --- |
| **— Sugarcane Bagasse —** |  |  |  |  |  |  |  |  |
| Pretreatment | 24085.77 | 14866.01 | 109.32 | 9219.77 | 40367 | 30785 | 0.43 | 0.034 |
| Enzyme hydrolysis | 17737.81 | 11789.29 | 59.46 | 5948.52 | 154462 | 27553 | 0.15 | 1.335 |
| Fermentation | 28571.03 | 16016.30 | 105.23 | 12554.73 | 440294 | 150768 | 0.25 | 0.708 |
| LA purification | 16552.62 | 8563.11 | 95.91 | 7989.51 | 590389 | 16481 | 0.03 | 0.498 |
| LA→Lactide conversion | 8689.82 | 7876.74 | 12.64 | 813.07 | 116406 | 26142 | 0.18 | 0.223 |
| Lactide purification | 8008.40 | 7205.70 | 11.79 | 802.70 | 127721 | 7451 | 0.05 | 0.229 |
| Polymerization | 8187.37 | 8061.22 | 2.13 | 126.15 | 19987 | 7161 | 0.26 | 0.006 |
| PLA purification | 8813.94 | 7008.93 | 26.53 | 1805.00 | 269045 | 8165 | 0.03 | 0.222 |
| Xylitol pretreatment | 7958.81 | 6641.21 | 21.86 | 1317.60 | 103945 | 24707 | 0.19 | 0.523 |
| Xylitol fermentation | 7362.82 | 3366.53 | 138.02 | 3996.30 | 398173 | 50615 | 0.11 | 5.490 |
| Xylitol purification | 3413.87 | 2334.33 | 53.45 | 1079.54 | 199825 | 32852 | 0.13 | 2.998 |
| AD purification | 10110.15 | 5728.99 | 90.05 | 4381.16 | 69196 | 12281 | 0.15 | 1.238 |
| AD activation | 5742.49 | 5729.00 | 5.48 | 13.49 | 406 | 9051 | 0.95 | 0.000 |
| AD methylation | 5784.06 | 5774.83 | 5.12 | 9.23 | 293 | 9099 | 0.97 | 0.000 |
| AD synthesis | 15654.76 | 15011.55 | 9.53 | 643.21 | 11047 | 13540 | 0.55 | 0.069 |
| **— Corn Stover —** |  |  |  |  |  |  |  |  |
| Pretreatment | 22165.71 | 14001.17 | 97.41 | 8164.53 | 51740 | 34687 | 0.40 | 0.034 |
| Enzyme hydrolysis | 16776.27 | 11261.65 | 57.20 | 5514.62 | 160604 | 26993 | 0.14 | 1.335 |
| Fermentation | 27338.27 | 15878.64 | 96.56 | 11459.62 | 436518 | 147537 | 0.25 | 0.708 |
| LA purification | 16411.05 | 8511.01 | 95.29 | 7900.04 | 606270 | 16128 | 0.03 | 0.498 |
| LA→Lactide conversion | 8636.84 | 7821.99 | 12.66 | 814.85 | 120743 | 26033 | 0.18 | 0.223 |
| Lactide purification | 7947.12 | 7155.72 | 11.69 | 791.40 | 130454 | 7420 | 0.05 | 0.229 |
| Polymerization | 8130.58 | 8005.30 | 2.11 | 125.28 | 20536 | 7131 | 0.26 | 0.006 |
| PLA purification | 8752.79 | 6960.32 | 26.51 | 1792.48 | 276316 | 8131 | 0.03 | 0.222 |
| Xylitol pretreatment | 7375.96 | 6078.69 | 26.09 | 1297.27 | 73087 | 24151 | 0.18 | 1.092 |
| Xylitol fermentation | 6853.58 | 3254.65 | 123.97 | 3598.93 | 383401 | 46424 | 0.11 | 5.490 |
| Xylitol purification | 3295.17 | 1447.22 | 136.55 | 1847.96 | 442852 | 30805 | 0.06 | 2.998 |
| AD purification | 9318.65 | 4864.39 | 105.65 | 4454.26 | 94876 | 14594 | 0.13 | 1.238 |
| AD activation | 4885.49 | 4864.39 | 5.83 | 21.10 | 927 | 11524 | 0.93 | 0.000 |
| AD methylation | 4915.67 | 4903.29 | 5.34 | 12.38 | 574 | 11548 | 0.95 | 0.000 |
| AD synthesis | 13304.51 | 12740.76 | 9.47 | 563.75 | 12925 | 14726 | 0.53 | 0.069 |

*rc = relative cost difference; Ex_D = exergy destruction; C_D = cost rate of exergy destruction; Ż = capital cost rate; f_c = exergoeconomic factor; IP = improvement potential.*

**SI.9 - Unit Fuel Exergy Cost (c_f)**

Table SI.9 presents the input quantities, unit prices, associated exergy flows, and resulting unit fuel exergy costs (c_f in USD/GJ) for the major process steps, reported for both sugarcane bagasse and corn stover. Values are calculated per the exergoeconomic formulation of Amid et al. (2021).

| **Process Step** | **Input** | **Bagasse Quantity (kg or kWh/batch)** | **Corn Stover Quantity (kg or kWh/batch)** | **Unit Price (USD/unit)** | **Sum Exergy (GJ/batch)** | **c_f (USD/GJ) Bagasse \| Corn Stover** |
| --- | --- | --- | --- | --- | --- | --- |
| **Pretreatment** | NaOH (kg) | 78,264 | 95,486 | 0.527 | 24,086 \| 22,166 | **4.38 \| 6.34** |
|  | Water (kg) | 3,834,944 | 4,678,821 | 0.002 | — | **—** |
|  | Electricity (kWh) | 14,841 | 18,104 | 0.191 | — | **—** |
|  | Heat (kWh) | 378,053 | 461,244 | 0.038 | — | **—** |
| **Enzyme hydrolysis** | Water (kg) | 3,092,419 | 2,988,313 | 0.002 | 17,738 \| 16,776 | **25.97 \| 29.12** |
|  | Electricity (kWh) | 2,764 | 2,663 | 0.191 | — | **—** |
|  | Heat (kWh) | 98,699 | 95,376 | 0.038 | — | **—** |
|  | Enzyme (kg) | 104,474 | 100,957 | 3.000 | — | **—** |
| **Fermentation** | CLS (kg) | 150,125 | 144,800 | 0.335 | 28,571 \| 27,338 | **35.07 \| 38.09** |
|  | CaCO₃ (kg) | 219,695 | 211,903 | 0.101 | — | **—** |
| **LA purification** | Water (kg) | 40,541 | 39,103 | 0.002 | 16,553 \| 16,411 | **73.90 \| 76.74** |
|  | Electricity (kWh) | 1,328 | 4,495 | 0.191 | — | **—** |
|  | HCl (kg) | 209,166 | 207,715 | 0.335 | — | **—** |
| **LA→Lactide** | Water (kg) | 3,844 | 3,817 | 0.002 | 8,690 \| 8,637 | **143.15 \| 148.18** |
|  | Heat (3 sub-steps, kWh) | 110,462 | 109,697 | 0.038 | — | **—** |
| **Lactide purification** | Ethyl acetate (kg) | 4,741 | 4,708 | 0.804 | 8,008 \| 7,947 | **159.12 \| 164.84** |
| **Polymerization** | Toluene (kg) | 22,425 | 22,269 | 0.670 | 8,187 \| 8,131 | **158.44 \| 163.93** |
|  | Zn-(Phe)₂ (kg) | 95.5 | 94.8 | 5.000 | — | **—** |
| **PLA purification** | Methanol (kg) | 32,616 | 32,390 | 0.268 | 8,814 \| 8,753 | **149.06 \| 154.15** |
| **SA pretreatment** | Xylose stream | 210,942 | 182,642 | — | 7,959 \| 7,376 | **78.89 \| 83.70** |
| **SA fermentation** | Yeast extract (kg) | 16,730 | 16,340 | 2.500 | 6,752 \| 6,319 | **99.64 \| 104.28** |
| **SA purification** | HCl + Activated C. | — | — | 0.335/0.90 | 3,912 \| 3,507 | **185.10 \| 201.75** |
| **Xylitol pretreatment** | Xylose stream | 210,942 | 182,642 | — | 7,959 \| 7,376 | **78.89 \| 83.70** |
| **Xylitol fermentation** | Rice bran ext. (kg) | 133,830 | 130,690 | 3.150 | 7,363 \| 6,854 | **100.53 \| 116.24** |
| **Xylitol purification** | Activated C. + Heat | — | — | 0.90/0.038 | 3,414 \| 3,295 | **187.70 \| 187.99** |
| **AD activation** | Heat (kWh) | 24,003 | 24,003 | 0.038 | 5,742 \| 4,885 | **30.11 \| 43.91** |
| **AD methylation** | Formaldehyde (kg) | 2,559 | 2,559 | 0.335 | 5,784 \| 4,916 | **31.71 \| 46.32** |
| **AD synthesis** | Phenol (kg) + HCHO | 225,427 | 191,500 | 0.11/0.34 | 15,655 \| 13,305 | **17.18 \| 22.93** |

*c_f = unit fuel exergy cost (USD/GJ), calculated as Σ(cost_input)/Σ(exergy_input) per Amid et al. (2021). Prices: electricity 0.191 USD/kWh (Australian grid); heat 0.038 USD/kWh; NaOH 0.527 USD/kg; HCl 0.335 USD/kg; CLS 0.335 USD/kg; enzyme 3.00 USD/kg; Zn-(Phe)₂ 5.00 USD/kg. Values in this table are reported per production batch (≈342,930 kg PLA per batch, equivalent to 342.93 FU), consistent with the plant's annual capacity of 32,500 t LA/year over 74.48 batches/year. Dividing by 342.93 converts these values to the FU basis (1000 kg PLA) used in Table 1 and Table 2. Individual unit fuel exergy values (Ex_in) are reported for exergoeconomic accounting purposes and should not be summed to obtain the overall system fuel exergy input, because exergy streams are transferred between successive process units and therefore appear as fuel exergy for more than one unit.*

**SI.10- Exergy Improvement Potential Hotspots and Optimisation Strategies**

Table SI.10 ranks unit operations by exergy improvement potential (IP = (1 − φ) × Ex_D, GJ/FU) across the four biorefinery scenarios and both feedstocks, and identifies technically feasible optimisation strategies. Scenarios: Sc1 = PLA + Electricity + Succinic Acid; Sc2 = PLA + Electricity + Xylitol; Sc3 = PLA + Adhesive + Succinic Acid; Sc4 = PLA + Adhesive + Xylitol.

**Table SI.10. Exergy improvement potential hotspots and recommended optimisation strategies (applicable to both sugarcane bagasse and corn stover).**

| **Hotspot (by IP)** | **Priority Scenario(s)** | **Priority Level** | **Technically Feasible Optimisation Strategies** |
| --- | --- | --- | --- |
| Electricity generation | Sc1, Sc2 | **HIGH** | Pinch-based heat integration; steam pressure-level optimisation; flue-gas and condensate heat recovery; minimise temperature mismatch between source and sink. |
| SA fermentation | Sc1, Sc3 | **HIGH** | Increase product titer/yield to reduce water handling; staged or continuous operation; improve internal heat recovery. |
| SA purification | Sc1, Sc3 | **HIGH** | Multi-effect or heat-integrated evaporation; hybrid separation (pre-concentration + crystallisation); condenser and flash-steam heat recovery. |
| Xylitol fermentation | Sc2, Sc4 | **HIGH** | Increase titer/yield; reduce auxiliary utilities (aeration, agitation) through process intensification; cell recycle or immobilisation. |
| Xylitol purification | Sc2, Sc4 | **MEDIUM** | Heat integration around concentration and crystallisation; hybrid separation (membrane pre-concentration + polishing). |
| Enzymatic hydrolysis | All (secondary) | **MEDIUM** | Improve solids loading within operability limits; improved mixing and heat management; heat recovery from hot hydrolysate. |
| AD purification | Sc3, Sc4 | **MEDIUM** | Reduce water removal duty through upstream lignin pre-concentration; integrate heating/cooling with available hot process streams. |

*IP = improvement potential = (1 − φ) × Ex_D (GJ/FU). Strategies listed are thermodynamically motivated directions; economic and engineering feasibility requires site-specific assessment. Corn stover and bagasse exhibit the same qualitative hotspot hierarchy; quantitative IP values are slightly higher for bagasse in Sc1/Sc2 due to higher electricity-related irreversibilities.*

**SI. 11.** Exergy flow (Sankey) diagrams tracking exergy inputs, useful outputs, and local irreversibility (exergy destroyed, shown in red) across all process units for sugarcane bagasse-based PLA biorefineries: (a) Scenario 1, (b) Scenario 2, (c) Scenario 3, and (d) Scenario 4 for corn stover.

**a)**

**b)**

**c)**

**d)**
